# Topology-Guided Sparse Voltage Measurement Placement for Transient-Voltage Fault-Bus Localization: A Physically Governed Dual-System Evaluation

**DOI:** 10.3390/s26196203

**Published:** 2026-09-30

**Authors:** Jisheng Xing, Zhankun Wang, Yu Xu

**Affiliations:** College of Electrical and Information Engineering, Beihua University, Jilin 132021, China; jisheng_xing@beihua.edu.cn (J.X.); yeah788@proton.me (Z.W.)

**Keywords:** sparse voltage measurement, sensor placement, fault-bus localization, topology-guided placement, quotient graph, condition-level validation, fault diagnosis, smart grid

## Abstract

Sparse voltage measurements make fault-bus localization depend on measurement placement and the physical distinguishability of location labels. We evaluate a deterministic, label-independent topology-guided placement protocol with physically governed localization classes and leakage-resistant condition-level validation on IEEE39 and IEEE123. In the original base protocols, topology-guided CNN1D Top-1 accuracy exceeded the prespecified primary-random layouts by 4.55 and 1.67 percentage points, respectively, although both paired bus-by-condition intervals included zero. In the reviewer-driven 1701-event IEEE39 physical-condition extension, Top-1 was 0.3683 for topology-guided placement and 0.3564 for primary random; the paired difference was +0.0119 with a 95% interval of −0.0493 to 0.0898, which also included zero. Separately, complete enumeration of all 84 feasible IEEE39 k = 3 layouts ranked the topology-guided layout first in Top-1 accuracy, Macro-F1, and mean-hop error under the frozen CNN1D protocol. Load-shift tests produced near-chance Top-1 values, and resistance-shift performance was lower at 20 Ω than at 0.1 Ω. Fixed-capacity, classifier, noise, timing, confusion, and topology-error analyses further bounded the result. The evidence supports reproducible pre-training measurement planning within the tested simulations without establishing statistically certain or universal superiority.

## 1. Introduction

Fault diagnosis and location in transmission systems span conventional signal-, model-, and data-driven methods, as summarized by Raza et al. [1]. Synchronized measurements form a distinct fault-location research line [2], including precision transmission-line localization with AI and PMU data [3] and Gaussian-process regression for three-terminal lines using two-terminal PMUs [4]. Cable studies have also formulated localization and assessment as an optimization problem [5]. In distribution networks, ANN localization has been evaluated under distributed-generation integration and fault-impedance variation [6], while adaptive ANN relaying has addressed high-resistance transmission-line faults [7]. Broader machine-learning frameworks have jointly considered detection, isolation, classification, and localization [8,9].

Deep-learning studies include CNN analysis of wave-matrix images [10], deep CNN classification and location for PMU-equipped active distribution networks [11], and state-estimator-assisted CNN localization [12]. Combined CNN-LSTM models have been applied to transmission-line classification and localization [13], while attention-guided multi-task learning provides another joint diagnosis architecture [14]. Graph-based methods explicitly connect localization to network structure: graph formulations have been studied under sparse distribution-network measurements [15], graph convolutional networks under high distributed-generation penetration [16], and cost-sensitive graph neural networks for distribution localization [17]. Comparative machine-learning work has also examined adaptability under renewable-energy integration [18].

The adjacent transient-monitoring literature has explored compressive sensing and phase-diagram-based analysis for partial-discharge source monitoring [19], and a hybrid 1D-CNN/LSTM architecture has been reported for open-circuit diagnosis in a multilevel inverter [20]. These studies motivate sparse transient measurement and temporal representation, respectively, but address different fault mechanisms and are not treated as direct fault-bus-localization baselines here.

Fault diagnosis is the broader task of detecting, classifying, localizing, and assessing abnormal conditions. Fault-bus localization is a narrower spatial decision within that workflow: it asks which electrically distinguishable location best explains the observed transient. A recent system-of-systems survey situates AI-enabled fault and anomaly detection within maintenance and forecasting for power-electronic components and converters [21]. We use that study only to contextualize broader smart-grid monitoring and data-driven diagnosis, not as a direct sensor-placement or fault-bus-localization baseline.

Within this narrower task, sparse measurement placement is a sensing-design decision made before classifier training. The layout determines whether transients from distinct fault locations are sufficiently spatially distinguishable; a downstream classifier can exploit only the information exposed by the chosen measurements. In this paper, the smart-grid context is therefore limited to sensing, monitoring, and data-driven diagnosis. The evaluated systems are the conventional meshed IEEE39 transmission benchmark and the predominantly radial IEEE123 distribution feeder; no converter-dominated or renewable-rich benchmark is constructed, and no claim is made about renewable-converter fault behavior.

Measurement placement is a separate design problem. Reviews cover synchrophasor applications and optimal PMU placement [22], while foundational work established minimal PMU placement for power-system observability [23]. Fault-location observability has been treated directly through minimal PMU placement [24]. More recent studies integrate placement with learning or event-identification objectives, including enhanced feature selection for PMU-based classification and localization [25], sparse PMU placement for grid-event detection [26], and joint CNN localization and PMU placement [27]. These studies motivate the present comparison of a label-independent topology-guided layout with frozen random and structural controls; measured buses determine observability, while location labels determine whether the classification problem is physically identifiable.

Their flexibility does not remove experimental-design hazards. Random event splitting can place the same factorial fault condition in training and test sets at different buses. A model may then exploit condition-specific signatures rather than demonstrate generalization across fault type and duration. Similarly, reporting fold-level training runs as independent statistical replicates understates uncertainty because the folds collectively define one out-of-fold prediction for a seed. Distribution-shift evaluation is therefore treated separately from base-protocol cross-validation.

Physical labels add a second problem. A named feeder node can be rigidly tied to the source, lie behind a normally open branch, or be connected to another endpoint through a closed near-zero-impedance switch. Such names are useful for model construction but need not be distinct learnable fault locations. Retaining them as classes can create duplicated or absent responses. The defensible alternative is a quotient graph: audit electrical equivalence, select canonical representatives, quarantine noncanonical events logically, and evaluate localization error in graph hops between distinguishable classes.

Dual-system evidence is useful only if it respects domain differences. The IEEE39 work copy provides signed, cycle-synchronous line-voltage projections at 1/60 s and represents electromechanical dynamics. The IEEE123 MathWorks feeder provides physical phase-to-ground EMT voltages at 20 kHz. We do not pool these raw signals, transfer a trained model between systems, or compare absolute accuracy as a common difficulty scale. Instead, the same task definitions, placement controls, condition-level folds, and topology-aware metrics are applied independently.

**Figure 1:**
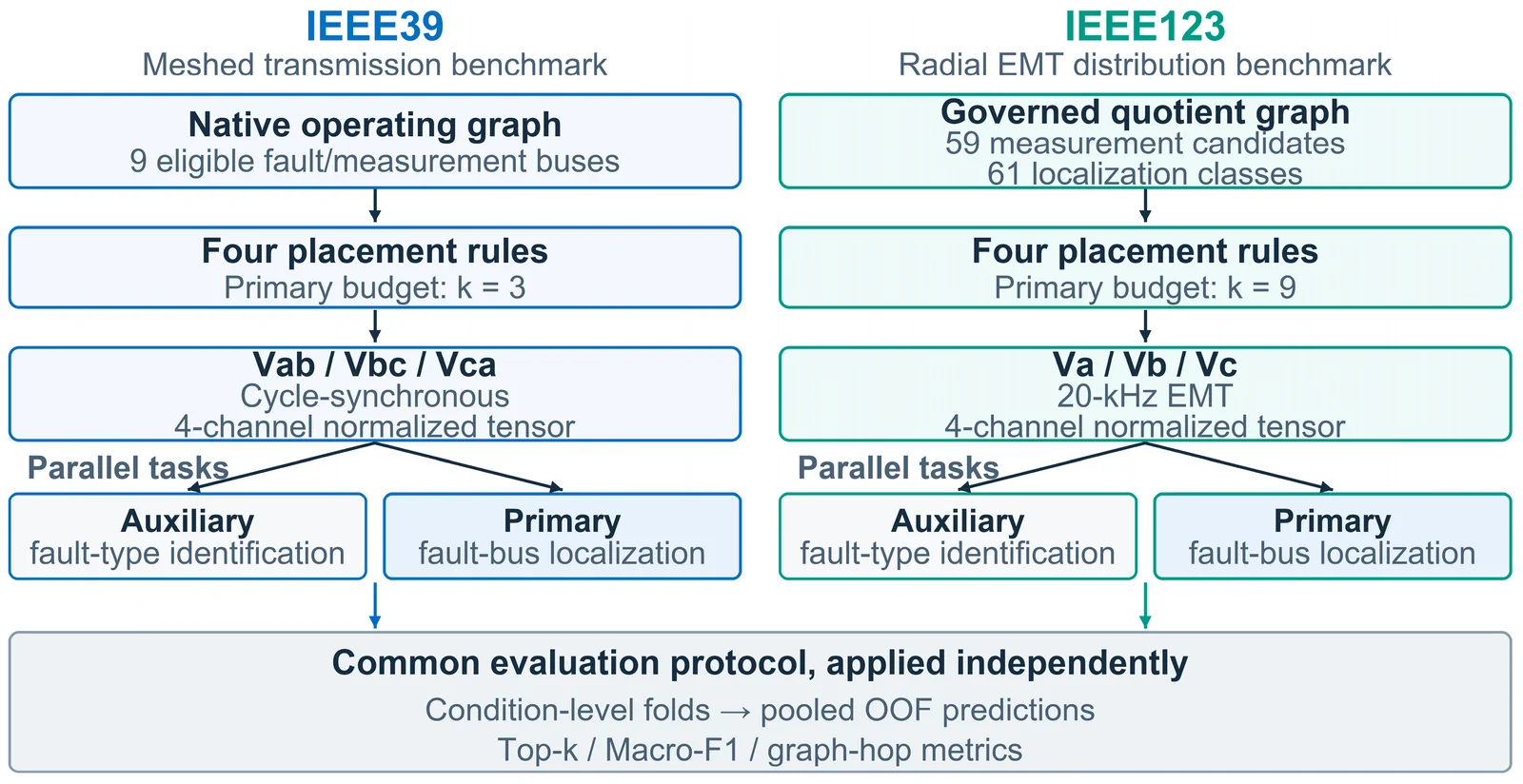
Parallel dual-system workflow. IEEE39 uses its native meshed graph, cycle-synchronous *V_ab_*/*V_bc_*/*V_ca_*, and k = 3; IEEE123 uses the governed quotient graph, 20 kHz EMT *V_a_*/*V_b_*/*V_c_*, and k = 9. In each independent branch, placement precedes classifier fitting; the auxiliary fault-type and primary localization tasks run in parallel, and neither true nor predicted auxiliary labels enter localization. Raw signals and trained models are not transferred.

Five gaps motivate this work: layouts are often fixed without comparison; evaluation is frequently limited to one system; event-random splits can leak factorial conditions; nominal node names are assumed to be valid labels; and placement conclusions are often evaluated with only one classifier and nominal measurements. We address these gaps through (i) a topology-only sparse placement protocol compared with frozen random and structural controls; (ii) quotient-graph governance that yields 61 electrically distinguishable IEEE123 labels; (iii) independent IEEE39 and IEEE123 evaluations under a common condition-level Latin-square design; (iv) complete OOF evaluation with paired bus-by-condition uncertainty; and (v) fixed-capacity, multi-classifier, measurement-noise, timing-sensitivity, confusion-matrix, and topology-error analyses. CNN1D remains an evaluation model rather than the claimed algorithmic innovation, and the placement rule is classifier-independent in construction. Section 2 defines the systems, labels, placement methods, models, perturbations, and statistics; Section 3 reports base, ablation, robustness, and shift results; Section 4 discusses interpretation and limits; and Section 5 concludes. The parallel dual-system workflow is summarized in Figure 1.

Figure 1 is read from top to bottom: physical network; governance and eligible measurement space; prespecified measurement placement; signal preprocessing; parallel auxiliary fault-type and primary fault-bus tasks; condition-level OOF validation; and topology-aware evaluation. Placement occurs before classifier evaluation, and the topology-guided rule reads neither localization labels nor validation scores. The auxiliary branch does not provide true or predicted fault-type labels to localization. IEEE39 and IEEE123 are processed independently: raw signals are not pooled, trained models are not transferred, and absolute accuracy is not treated as a common difficulty scale.

## 2. Materials and Methods

### 2.1. Problem Definition

An event contains a time vector, voltages from the frozen k-sensor layout, a seven-class grounded fault-type label, a system-specific location label, and a four-channel-per-sensor temporal input tensor. The auxiliary fault-type classifier uses summary features. Independently, the primary localization classifier ranks fault-location classes from the full temporal tensor and receives neither the true nor predicted fault type.

For ranked class probabilities, Top-k is the fraction of events whose true class lies among the k largest probabilities. Macro-F1 is the unweighted classwise mean. On the quotient/native evaluation graph, shortest-hop distance compares predicted and true locations; within-one and within-two report distances no greater than one and two hops, respectively.

### 2.2. Dual-System Models and Signals

The frozen physical and dataset definitions are summarized in Table 1.

IEEE39 retains the native fixed-step setting *T_s_* = 1/60 s. A five-second no-fault run was required to show bounded generator speed, relative rotor-angle, and line-voltage projection trends before fault events were accepted. The saved event signal contains *V_ab_*, *V_bc_*, and *V_ca_* at 121 points from 0 to 2 s. These are signed cycle-synchronous line-voltage projections, not RMS values and not reconstructed 60 Hz EMT waveforms. The nine fault locations and nine candidate measurements are buses 3, 4, 15, 16, 18, 21, 23, 26, and 29. The New England system lineage is cited in [28].

IEEE123 uses the official MathWorks Simscape Electrical IEEE 123 Node Test Feeder EMT project in MATLAB R2025b [29]. Direct audit of all 1281 qualified IEEE123 event files found 6401 samples from 0 to 0.32 s at 50 µs. Raw outputs are physical *V_a_*, *V_b_*, and *V_c_*. All formal events use the nominal 100% load condition (L100). The final data contain 61 electrically distinguishable fault-location classes and 59 independent measurement candidates. The model supports seven grounded faults; pure line-to-line and ungrounded three-phase faults are outside the scope. IEEE39 is treated as a conventional meshed transmission benchmark, whereas IEEE123 is treated as a predominantly radial distribution-feeder benchmark. The present study does not explicitly model a converter-dominated renewable-energy network.

### 2.3. Fault Scenarios

Both systems use A–G, B–G, C–G, A–B–G, A–C–G, B–C–G, and A–B–C–G faults. Each is combined with durations of 4, 6, and 12 nominal cycles, creating 21 conditions per location. IEEE39 therefore contains 9 × 7 × 3 = 189 events and IEEE123 contains 61 × 7 × 3 = 1281 events. The systems retain their own resistance semantics rather than asserting cross-system equality.

**Figure 2:**
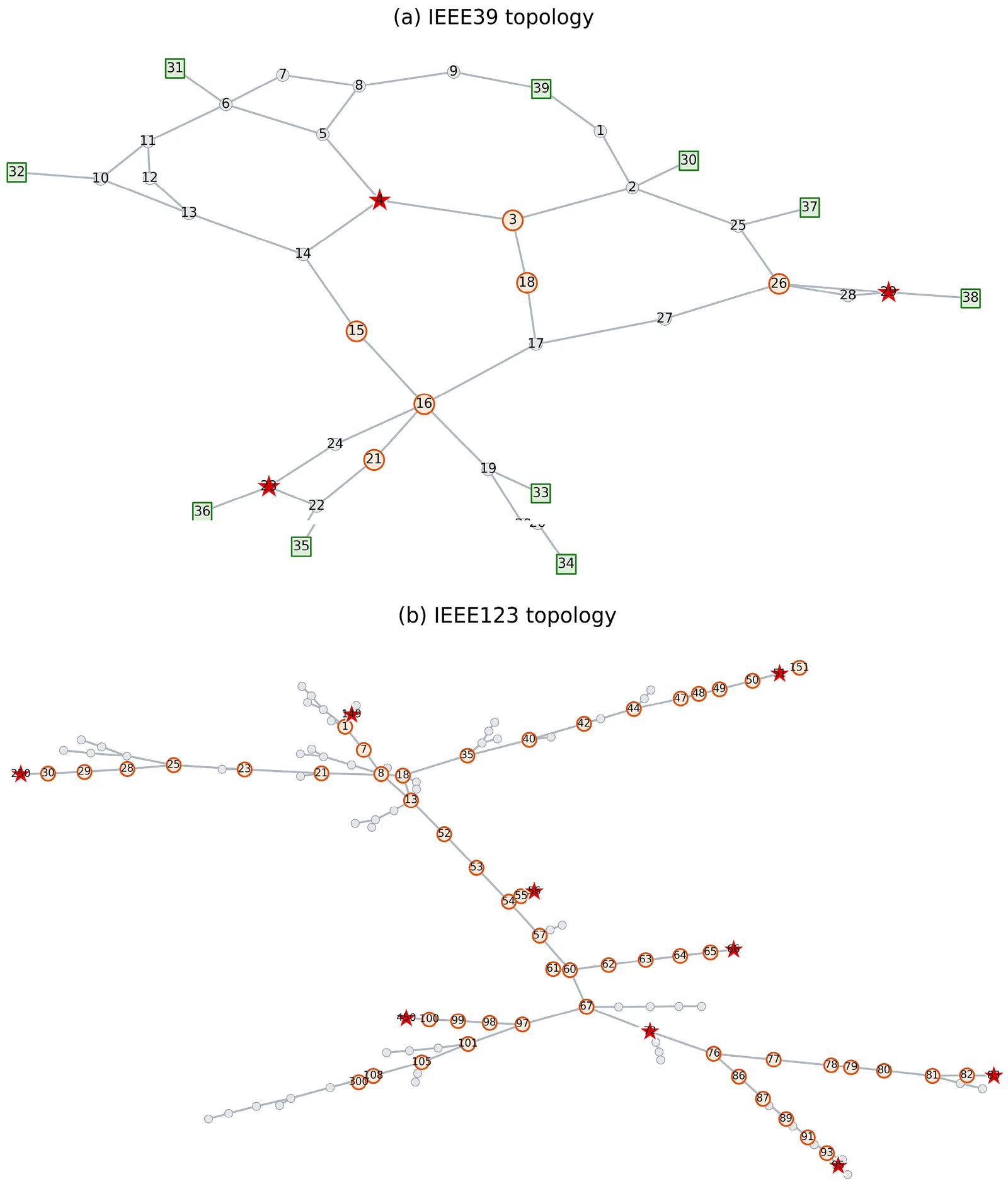
Actual connectivity graphs used in the study. (**a**) IEEE39 meshed transmission topology: generator/source buses are boxed, the nine eligible fault/measurement buses are outlined, and the primary k = 3 topology-guided sensors are starred. (**b**) The energized IEEE123 feeder: the 61 quotient-governed localization classes are distinguished from gray context nodes, and the primary k = 9 topology-guided measurement sensors are starred.

For IEEE39, faults start at 1.0 s and clear after 4, 6, or 12 cycles; records end at 2.0 s. The work-copy fault blocks retain *R_pn_* = 1 Ω and *R_ng_* = 1 Ω. For IEEE123, direct reads of all 1281 formal MAT files establish fault inception at 0.06 s. Four cycles clear at 0.12665 s with 1333 active samples; six cycles clear at 0.16 s with 2000 samples; and twelve cycles clear at 0.26 s with 4000 samples. Raw records end at 0.32 s, leaving 1201 samples from twelve-cycle clearance through record end. IEEE123 retains *R*_on_ = 0.001 Ω, *R_g_* = 0.001 Ω, and *G*_off_ = 10^−5^ S; these parameters are not asserted to be cross-system equivalent. The actual network connectivity and the primary topology-guided sensor locations for both systems are shown in Figure 2.

The original IEEE39 base protocol remains unchanged at 189 events: nine fault buses crossed with seven fault types and three durations at L100 and *R_pn_* = *R_ng_* = 1 Ω. The reviewer-driven physical-condition extension crosses the same buses, fault types, and durations with loads of 90%, 100%, and 110% and resistance levels of 0.1, 1, and 20 Ω, applied simultaneously to *R_pn_* and *R_ng_*. It contains 189 physical condition IDs across nine fault buses for 1701 events. The scalar resistance level is not described as total phase-to-ground resistance. All 1701 active MAT files passed the frozen integrity audit.

### 2.4. Signal Preprocessing

Preprocessing uses the same event-local, per-measurement-bus normalization rule in both systems. Let *V*_1_, *V*_2_, and *V*_3_ denote IEEE39 *V_ab_*/*V_bc_*/*V_ca_* or IEEE123 *V_a_*/*V_b_*/*V_c_*, respectively. The executed cache code defines $V_{\mathrm{vec}}(t)=\sqrt{V_{1}{\left(t\right)}^{2}+V_{2}{\left(t\right)}^{2}+V_{3}{\left(t\right)}^{2}}$ and *B* = median{*V*_vec_(*t*): *t* < *t*_fault_} using strictly prefault samples. Each sensor contributes the four dimensionless channels *V*_1_/*B*, *V*_2_/*B*, *V*_3_/*B*, and *V*_vec_/*B*. IEEE123 uses the inclusive interval from *t*_fault_ − 0.05 s to *t*_fault_ + 0.15 s (0.01–0.21 s because *t*_fault_ = 0.06 s, at 50 μs, 4001 points); four- and six-cycle clearances lie inside this crop, whereas 12-cycle cases are right-censored because clearance occurs at 0.26 s. IEEE39 retains its native 121-point *V_ab_*/*V_bc_*/*V_ca_* record. *V*_vec_ is a three-channel vector magnitude and is not described as IEEE39 RMS.

The tensor is ordered as event × sensor × four channels × time and flattened to event × 4 k × time for CNN1D. Thus k = 3, 6, 9 produce 12, 24, 36 input channels in the existing architecture. For SVM, XGBoost, and MLP, each normalized channel is deterministically summarized by temporal mean, standard deviation, minimum, maximum, and root-mean-square value. StandardScaler is fit only on the relevant outer-training fold. The comparison is a placement-tendency check, not a strict algorithm leaderboard, because CNN1D receives full temporal sequences while the other classifiers receive summary features.

### 2.5. IEEE123 Quotient-Graph Label Governance

Let the frozen qualified operating graph be defined by Equation (1). The equivalence relation in Equation (2) holds when two named nodes must not be treated as independent localization labels under prespecified physical governance: rigid/source equivalence, membership in the energized component, and a closed near-zero-impedance switching relation. It is never inferred from CNN accuracy or waveform similarity. Equations (3)–(5) define each equivalence class, the quotient-node set, and the quotient graph.

*G* = (*V*, *E*) (1)

*u* ∼ *v* (2)

[*u*]_∼_ = {*v* ∈ *V*: *v* ∼ *u*} (3)

*V*/∼ = {[*v*]_∼_: *v* ∈ *V*} (4)

*G*/∼ = (*V*/∼, *E*/∼) (5)

A quotient edge connects two distinct classes if and only if at least one valid energized branch in the original qualified graph crosses between their members. Ordinary nonzero-impedance lines are retained, open switches are not closed, and no self-loop created by contraction is retained.

*q*: *V* → *V*/∼, *q*(*v*) = [*v*]_∼_ (6)

Equation (6) defines the deterministic mapping q from raw nodes to quotient classes. For a raw bus label y, the governed localization label is given by Equation (7).

*y_q_* = *q*(*y*) (7)

**Figure 3:**
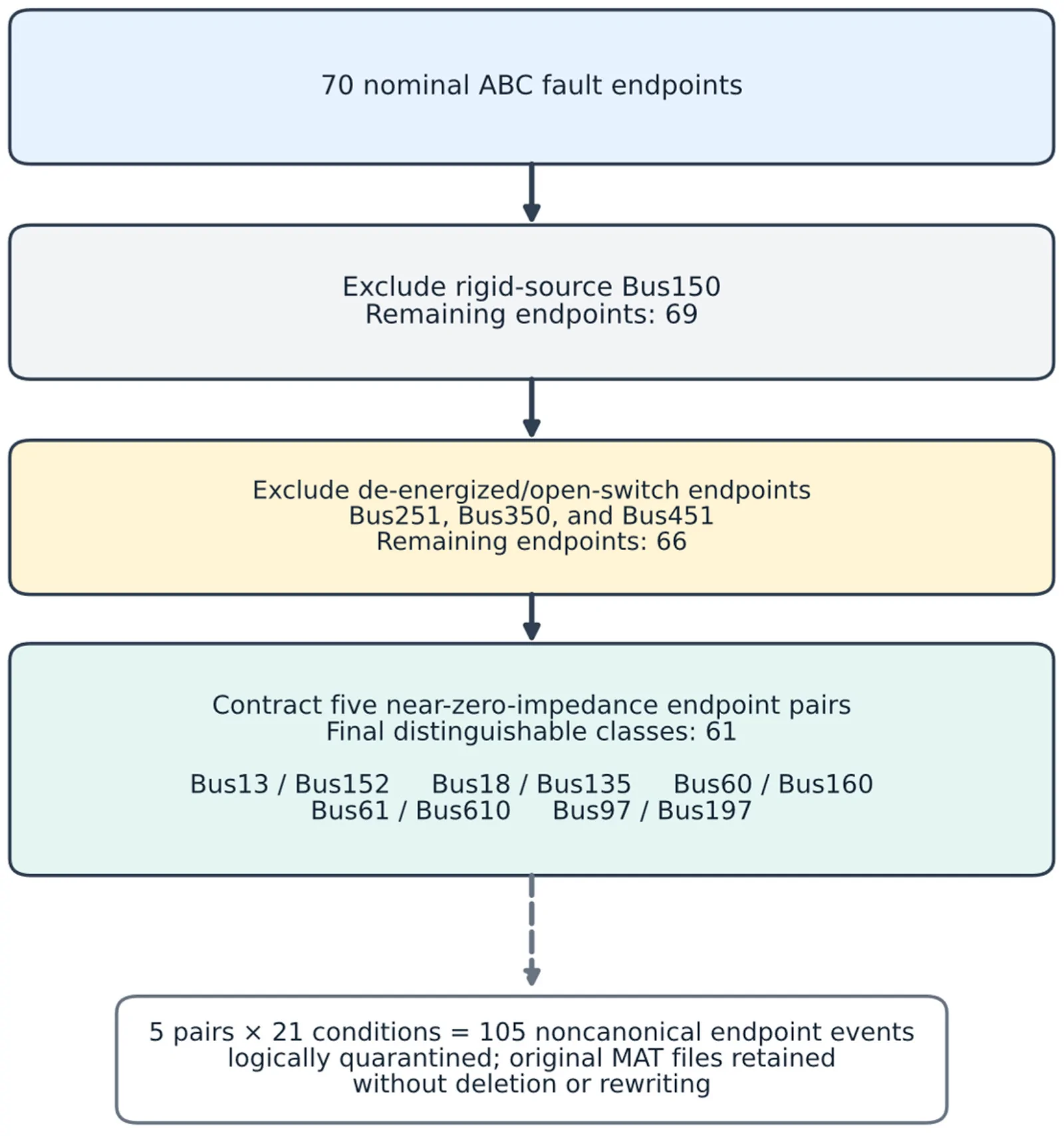
Pre-training, performance-independent IEEE123 label governance. Original MAT files are neither deleted nor rewritten; ordinary nonzero-impedance lines are not contracted and open switches are not closed. The resulting 61 classes are electrically distinguishable classes in the energized main component, not an arbitrary selection of 61 named buses.

For IEEE123, Bus150 is rejected as an unsupported rigid-source label; buses 251, 350, and 451 lie outside the energized component. The five frozen contractions are 13∼152, 97∼197, 18∼135, 60∼160, and 61∼610, with canonical representatives 13, 97, 18, 60, and 61. The 105 noncanonical endpoint events are preserved in the source archive but excluded from the final 61-class analysis dataset. Quotient edges and all hop metrics use the governed graph. Figure 3 summarizes this pre-training sequence, and Algorithm 1 lists the deterministic quotient-graph construction steps.
**Algorithm 1.** Physics-governed quotient-graph construction.**Item****Pre-Training Operation**InputOriginal graph G; energized-state information; switch state/impedance; candidate localization labels.1Determine the energized component under the frozen operating topology.2Remove the unsupported rigid-source label.3Exclude de-energized or normally open isolated nodes.4Form frozen equivalence classes using only physical connectivity and switching rules.5Choose one deterministic canonical representative per class.6Construct quotient edges from valid branches crossing distinct classes.7Generate q and map raw labels to governed labels.8Preserve but quarantine noncanonical source events.OutputQuotient graph *G*/∼, mapping q, and valid localization classes.

### 2.6. Measurement Layout Methods

Let *M* be the frozen set of physically logged and extractable measurement candidates, *r* the governed source/reference node used for deterministic initialization, *S_t_* the selected set after t sensors, and *d_G_*(*u*, *v*) the shortest-path distance on the governed operating graph. The measurement budget is *k*. Equation (8) selects *r* when *r* is eligible, otherwise it selects the first nearest eligible candidate under the frozen candidate order. Subsequent sensors maximize the minimum distance to the selected set by Equation (9), and Equation (10) updates the set.

(8) $$s_{1}=r,\;r\in M;\;s_{1}=\arg\underset{v\in M}{\min}d_{G}\left(r,v\right),\;r\notin M$$

(9) $$s_{t}=\arg\underset{v\in M\setminus S_{t-1}}{\max}\underset{u\in S_{t-1}}{\min}d_{G}\left(v,u\right)$$

*S_t_* = *S_t_*_−1_ ∪ {*s_t_*} (10)

Ties use the frozen candidate order. No fault label, classifier, training accuracy, validation score, or post hoc significance result enters placement selection. The graph-distance matrix is computed once before the greedy iterations.

Distance-dispersion belongs to the same broad farthest-first/max-min family but uses a different geometry. It maps candidates to their scalar distance from the source, initializes with the first minimum and first maximum coordinate, then maximizes minimum absolute separation on that one-dimensional coordinate. Branches with equal source distances are indistinguishable to this objective even when mutually distant in the graph. Degree-centrality ranks unweighted degree with stable candidate-order ties. Random layouts sample uniformly without replacement using MATLAB mt19937ar and a frozen seed equal to the base layout seed plus 100k. The primary random was fixed by protocol before model evaluation and was not selected for weak performance; every additional random layout is reported as a distribution.

The four controls answer different design questions. Topology-guided placement is a deterministic structural planning heuristic that uses full pairwise graph geometry. Distance-dispersion retains only scalar source distance, so candidates at equal source distances can be equivalent to its objective even when they lie on different branches. Degree-centrality tests whether locally well-connected nodes alone yield useful sensing positions. Primary random is a prespecified non-structural comparator whose seed and selected buses were frozen before performance evaluation. No fault waveform, classification label, training accuracy, validation result, or statistical-significance result enters any selection step. Accordingly, topology-guided placement is a reproducible topology-based placement rule, not an optimality claim.

The main budgets are k = 3 for IEEE39 and k = 9 for IEEE123, corresponding to 3/39 = 7.69% and 9/123 = 7.32% of named system nodes. The IEEE123 density denominator is 123 named nodes, not 61 fault classes; the 59 independent measurement candidates are reported separately. Budget analyses at k = 3, 6, 9 are descriptive because the CNN’s first layer expands with input channels. The four prespecified placement controls are summarized in Table 2.

### 2.7. Auxiliary Fault-Type Identification Method

Fault-type identification is an auxiliary seven-class task. A balanced multinomial logistic-regression classifier uses the mean, standard deviation, minimum, and maximum of every normalized channel. A StandardScaler and classifier are fitted within each training fold; maximum iterations are 2000. Within an outer-training partition, fivefold training-only cross-validation produces out-of-fold class probabilities when exploratory conditioning requires them, followed by a fit on the complete outer-training partition and one prediction on the outer test. The task is evaluated with accuracy and Macro-F1. Its output is not passed to the localization classifier, so fault-type-task saturation cannot be presented as evidence for localization-placement gains.

### 2.8. Primary Fault-Bus Localization Classifier

The primary fault-bus localization model is a compact one-dimensional temporal CNN chosen for consistency across the two systems; it is not proposed as a novel architecture. Its input has 4*k* channels. Conv1 applies 64 filters of width 9, stride 2, and padding 4, followed by ReLU and dropout 0.1. Conv2 applies 64 filters of width 7, stride 2, and padding 3, followed by ReLU. Adaptive average pooling produces 64 × 8 outputs; the flattened 512 features are mapped to C location classes, where *C* = 9 for IEEE39 and *C* = 61 for IEEE123. Training uses cross-entropy and Adam with a learning rate of 0.001.

Hyperparameters are selected only within the outer-training data by two inner condition folds. The final epoch count is the rounded median of inner best epochs and is then fixed for outer training; no outer-test early stopping occurs. The outer test is evaluated once. Seeds 2026–2030 define the five formal pooled-OOF replicates. At the main budgets, the parameter counts are 40,329 for IEEE39 *k* = 3 and 80,829 for IEEE123 *k* = 9; counts change with k. CNN1D never receives auxiliary fault-type probabilities or the true fault type. Training was full-batch (no DataLoader minibatch). Adam used the PyTorch 2.9 defaults *β*_1_ = 0.9, *β*_2_ = 0.999, *ε* = 10^−8^, and weight decay 0. Inner searches allowed at most 200 epochs with patience 25; the final epoch was the rounded median of the two inner best epochs. The Python 3.14.2 execution environment controlled the Python and NumPy random states [30], and PyTorch 2.9 controlled the CNN1D optimizer and random state [31]; execution was CPU-only. The auxiliary task used the documented scikit-learn LogisticRegression implementation [32]. The executed CNN1D architecture is summarized in Table 3.

### 2.9. Controlled Ablation and Classifier Comparisons

The fixed-capacity ablation uses a 36-channel input for every *k*. The frozen deterministic layout order fills the first k sensor blocks and all remaining sensor blocks are zero; hidden width and every trainable layer remain unchanged. Both systems use *k* = 3, 6, 9; the original outer folds; and seeds 2026–2030. The classifier-independence experiment uses the primary *k* = 3 IEEE39 and *k* = 9 IEEE123 topology and primary-random layouts with CNN1D, radial-basis SVM, XGBoost, and a two-layer MLP. CNN1D/MLP use five seeds; deterministic SVM/XGBoost each contribute one OOF estimate.

As an additional reviewer-driven layout-space analysis, the IEEE39 *k* = 3 experiment exhaustively evaluated all C(9,3) = 84 feasible measurement layouts. Every layout used the original 189-event condition-level protocol, the same three outer folds, seeds 2026–2030, identical CNN1D training rules, and the same preprocessing. The enumeration was complete rather than randomly sampled; no layout was added or removed according to its observed performance.

### 2.10. Measurement-Uncertainty Protocols

Models are trained only on clean data. At test time, zero-mean Gaussian noise is added at clean, 40, 30, and 20 dB; signal power and corresponding noise variance are computed separately for every event, sensor, and channel. For IEEE123, independent deterministic integer offsets are sampled per event/sensor from ±1, ±2, or ±5 samples and edge-padded without circular wrap. For IEEE39, independent offsets are sampled on the native cycle-synchronous grid at ±0.10*T_s_*, ±0.25*T_s_*, and ±0.50*T_s_* and applied by linear interpolation with edge clipping. The IEEE39 test is a synthetic timing-sensitivity experiment on the native representation, not a hardware PMU jitter model.

### 2.11. Condition-Level Latin-Square Validation

The factorial condition identifier combines seven fault types with three durations. Twenty-one condition IDs are assigned across three outer folds so that each fold contains seven conditions and balances type and duration. A condition ID is assigned to the same outer fold at every fault location. Consequently, evaluation withholds complete factorial conditions rather than randomly withholding events while leaving the same condition at other buses in training.

Each event enters the outer test exactly once per model seed. The three outer-test prediction files are concatenated to one complete OOF prediction before a seed-level metric is calculated. Five seeds therefore yield five statistical estimates, although 15 fold trainings are executed. The test fold never selects checkpoints, layouts, candidate labels, or preprocessing parameters.

### 2.12. Metrics and Statistical Analysis

Formal summaries compute Top-1, Top-3, Macro-F1, mean and median hop, within-one-hop, and within-two-hop for each seed’s complete OOF set. The mean ± SD uses five pooled-OOF seed estimates unless the classifier is deterministic. Fold trainings and time samples are not statistical replicates. Uniform Top-1 references are 1/9 for IEEE39 and 1/61 for IEEE123. The paired bus × condition bootstrap remains the sole primary inference for topology versus primary random; the controlled ablations are reported descriptively with prespecified seeds and no post hoc significance selection. The complete IEEE39 84-layout enumeration is treated as a finite candidate-space ranking rather than a replacement for the primary paired bus × condition uncertainty analysis.

Layout differences use the same event and seed. The primary uncertainty analysis independently resamples fault buses and condition IDs 1000 times, evaluates their Cartesian product, and retains paired predictions and all five seeds. IID-event, bus-cluster, and condition-cluster intervals are sensitivity analyses only. The earlier event-IID confidence interval is formally retired because it treats crossed factorial event cells as independent. No method was selected by its significance outcome.

### 2.13. Reviewer-Driven IEEE39 Extended Physical-Condition Evaluation

The extension uses the existing CNN1D and the frozen k = 3 topology-guided (buses 4, 23, and 29), primary-random (23, 29, and 3), and distance-dispersion (4, 23, and 15) layouts. A condition ID is the complete tuple of fault type, duration, load, and resistance. The 189 IDs are assigned to three frozen outer folds with 63 conditions per fold, and all nine fault-bus events for one condition enter the same fold. Seeds 2026–2030 yield five complete pooled-OOF estimates per layout; the 15 fold trainings are not treated as independent replicates.

Topology-guided minus primary-random uncertainty uses 5000 paired Cartesian-product bootstrap replicates. Fault buses and the 189 condition IDs are resampled independently with seed 2026, and paired predictions and all five model seeds are retained. Load shift trains only on L100 across all resistance levels and tests L90 and L110 separately. Resistance shift trains only at 1 Ω across all loads and tests 0.1 and 20 Ω separately. Test-factor events do not enter training, epoch selection, hyperparameter selection, or any fitted dataset-level normalization.

## 3. Results

### 3.1. Dataset and Label Integrity

The frozen manifests contain 189/189 IEEE39 and 1281/1281 IEEE123 events, exactly 21 conditions per location. Every condition ID maps to one outer fold across all locations, and every event occurs once in the complete OOF reconstruction. Dataset audits found no NaN/Inf values or abnormal complete-waveform duplicate groups in the qualified sets. Source-file hashes were retained; the quotient operation is a logical mapping and did not mutate original MAT files. Direct time-axis reads additionally verified 1281/1281 IEEE123 raw records at 0–0.32 s/6401 samples, with observed clearance and post-fault samples for all duration groups.

The IEEE123 governance sequence removed the unsupported Bus150 class, excluded three normally open island nodes, and contracted five near-zero-impedance endpoint pairs. The 105 noncanonical endpoint events are preserved in the source archive but excluded from the final 61-class analysis dataset. This process reduced the learnable inventory from a nominal intermediate set to 61 physically defensible labels and prevented the classifier from being rewarded or penalized for distinctions the model does not electrically support.

### 3.2. Auxiliary Fault-Type Identification

Fault-type identification is an auxiliary task. Table 4 reports complete pooled-OOF performance for the primary topology layouts and duration-shift ranges; detailed fault-type and duration-shift rows are in Supplementary Table S2. Placement effects were small relative to localization effects, and the auxiliary output is not passed to the localization classifier; no joint-model gain is claimed.

Macro-F1 is the unweighted mean of the seven classwise F1 values, so every fault type contributes equally after its precision and recall are combined. It complements overall accuracy, which reflects the aggregate proportion of correctly classified events. For IEEE123, base accuracy and base Macro-F1 are both 0.9961. Their agreement shows that the high base score is accompanied by consistently high classwise precision–recall balance rather than being driven only by a subset of classes. It does not establish arbitrary out-of-distribution fault-type robustness, solve the primary localization task, or imply that the auxiliary task guarantees localization.

For IEEE39, the base accuracy of 0.7566 and base Macro-F1 of 0.7561 are likewise close, showing only that aggregate classwise balance and overall correctness are similar in the base auxiliary task. Duration holdouts introduce substantially greater variation: IEEE123 accuracy spans 0.8384–1.0000 and Macro-F1 spans 0.8286–1.0000, while IEEE39 accuracy spans 0.5714–0.8254 and Macro-F1 spans 0.5595–0.8162. The variation is especially pronounced for IEEE39, demonstrating sensitivity of auxiliary fault-type identification to withheld temporal conditions. The auxiliary fault-type classifier is diagnostic rather than hierarchical: neither its true nor predicted fault-type label is supplied to the primary fault-bus localization CNN1D.

### 3.3. CNN1D Main Results

The main topology-guided CNN1D results are reported in Table 5.

**Figure 4:**
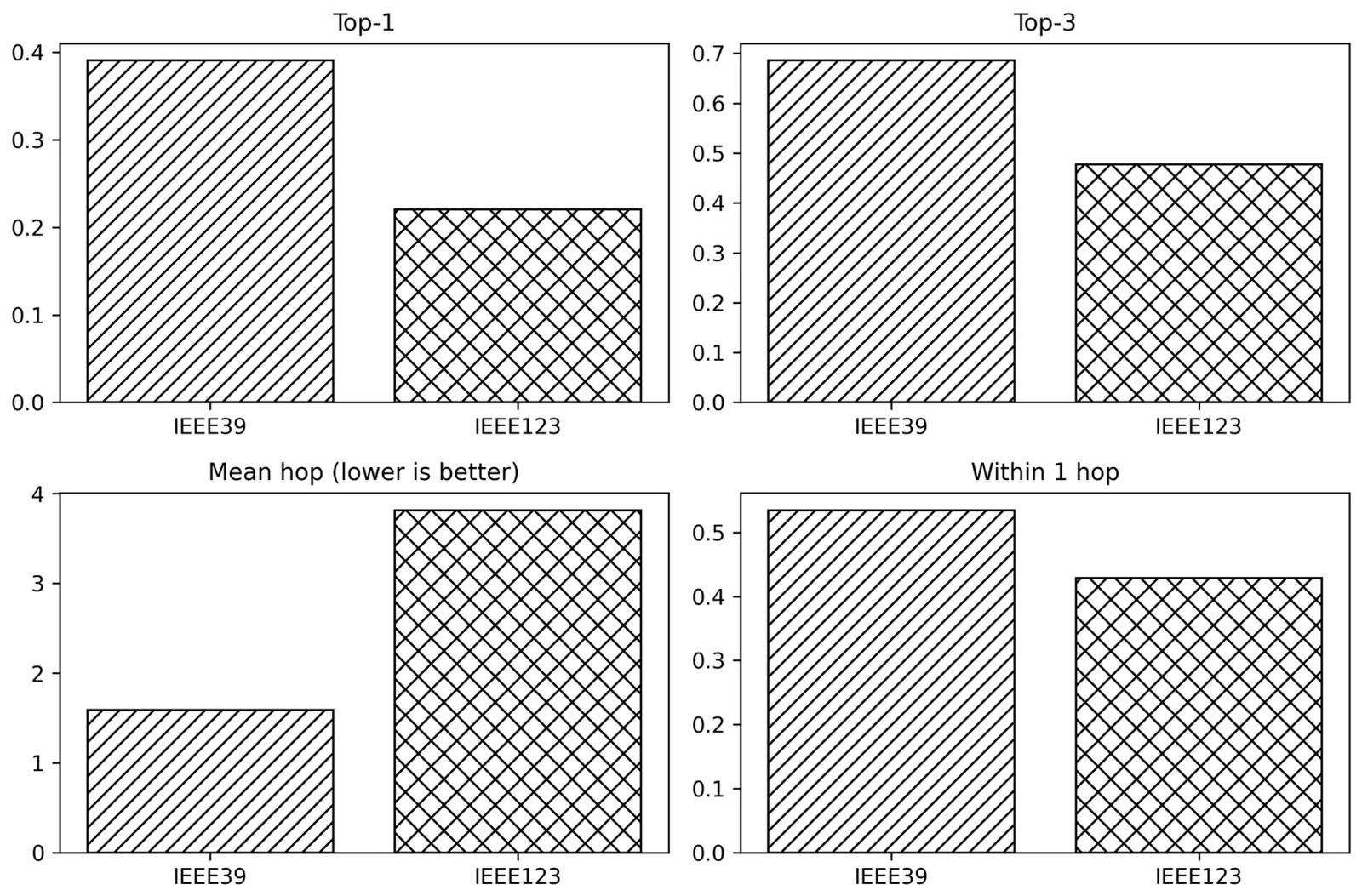
Exact-label and topology-aware pooled-OOF metrics for the primary topology-guided layouts; mean hop is lower-is-better.

The topology-guided pooled-OOF Top-1 is approximately 0.390 for IEEE39 and 0.220 for IEEE123 under the corrected five-seed aggregation. Top-3 and hop-based metrics are materially higher than exact Top-1, showing that some errors retain useful regional information. These values do not constitute high-accuracy direct protection. They describe candidate-bus prioritization and fault-region narrowing in two simulation studies; operating protection still requires deterministic timing, dependability, security, and hardware validation. The principal exact-label and hop-aware results are visualized in Figure 4.

The absolute IEEE39 and IEEE123 values are not compared as a shared difficulty measure. They differ in class count, signal physics, record length, topology, and candidate density. The appropriate dual-system comparison is whether a protocol direction recurs under each system’s native processing, accompanied by its uncertainty.

### 3.4. Layout Comparison and Corrected Uncertainty

The absolute placement-method results are reported in Table 6. The paired bus-by-condition bootstrap results are reported in Table 7.

Relative to primary random, topology’s Top-1 point difference is +0.0455 for IEEE39 (95% CI −0.0296 to 0.1397) and +0.0167 for IEEE123 (95% CI −0.0180 to 0.0567). Both include zero under the sole primary bus × condition analysis. The earlier event-IID confidence intervals were over-narrow and were retired.

**Figure 5:**
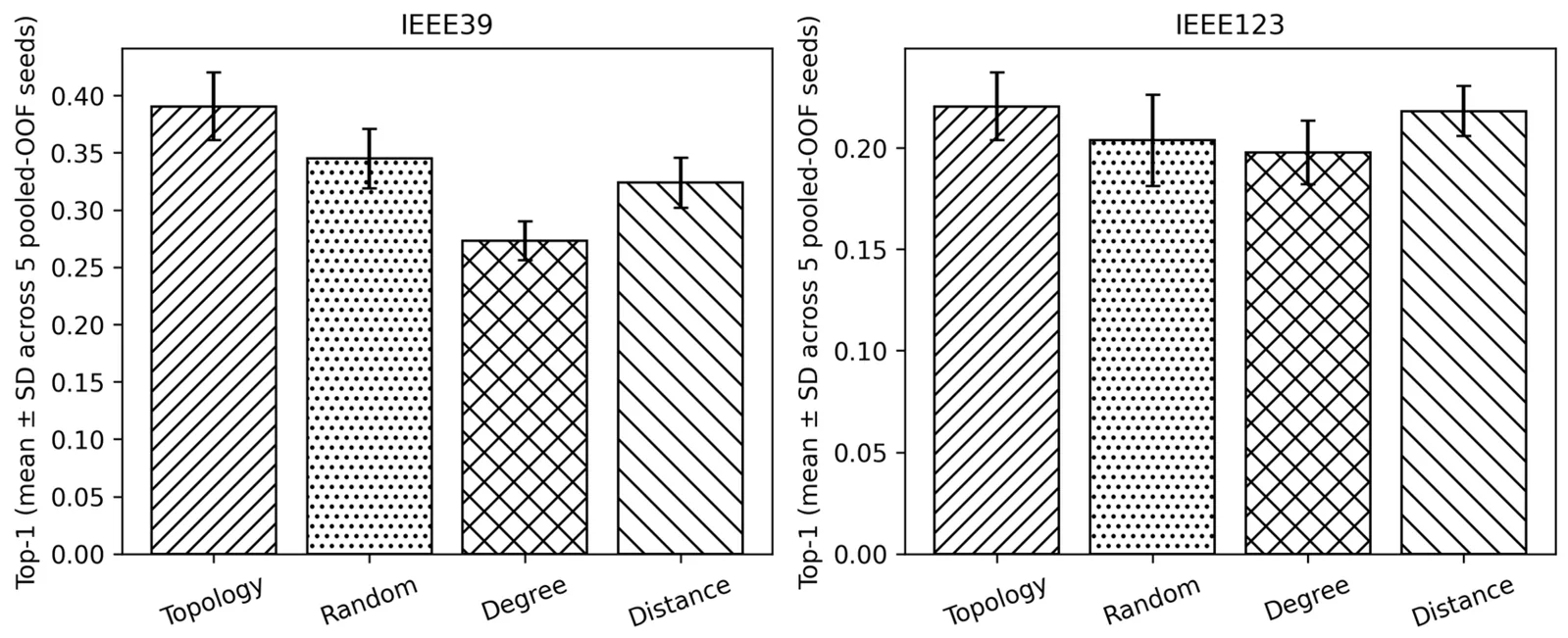
Four-layout Top-1 comparison. Error bars are the SD across five pooled-OOF model-seed estimates, not across the 15 fold trainings.

Topology-guided placement showed higher point estimates than degree-centrality on selected metrics, including IEEE39 Top-1, Macro-F1, and mean hop and IEEE123 mean hop. Against distance-dispersion, evidence is weaker: IEEE123 shows no statistically supported difference, while IEEE39 primarily favors topology in mean hop. The appropriate claim is a positive but uncertain base-distribution point advantage over the primary random and selected structural point advantages over degree—not comprehensive superiority. The four-layout Top-1 comparison is shown in Figure 5.

### 3.5. Frozen Random-Layout Distribution

**Figure 6:**
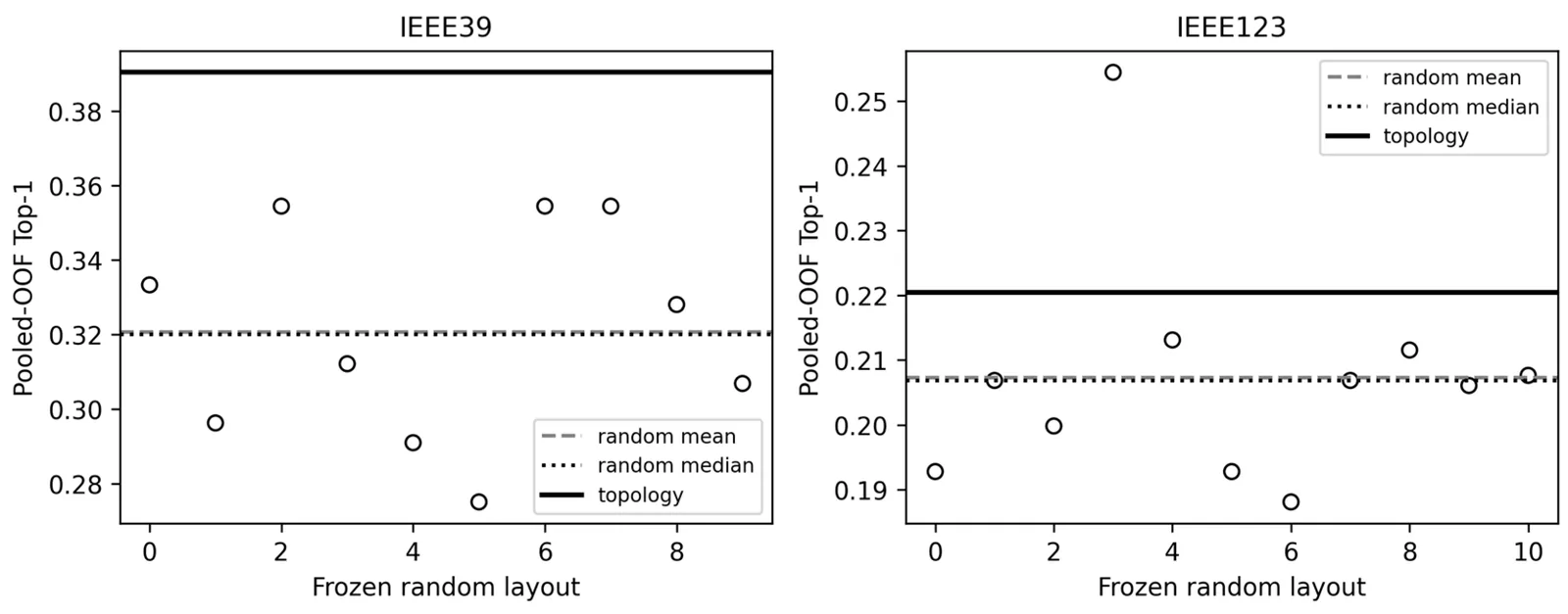
Complete-OOF performance of every frozen random layout at the main budget, with topology, random mean, and random median reference lines.

All random layouts frozen before evaluation are retained. IEEE39: n = 10 unique layouts, mean = 0.321, median = 0.320, range = 0.275–0.354, topology percentile = 100.0%, plus-one empirical *p* = 0.091, minimum resolution = 0.091; IEEE123: n = 11 unique layouts, mean = 0.207, median = 0.207, range = 0.188–0.254, topology percentile = 90.9%, plus-one empirical *p* = 0.167, minimum resolution = 0.083. The best random draw is not substituted post hoc for the protocol-frozen primary random. The distribution of all frozen random layouts is shown in Figure 6.

Because the IEEE39 candidate set contains only nine eligible buses at k = 3, all 84 feasible layouts were subsequently evaluated under the frozen base protocol. The topology-guided layout ranked 1st/84 for Top-1 accuracy, 1st/84 for Macro-F1, and 1st/84 for mean-hop error. This is an exact ranking within the finite IEEE39 candidate space under the fixed CNN1D protocol rather than evidence of universal optimality.

### 3.6. Measurement-Budget Sensitivity

**Figure 7:**
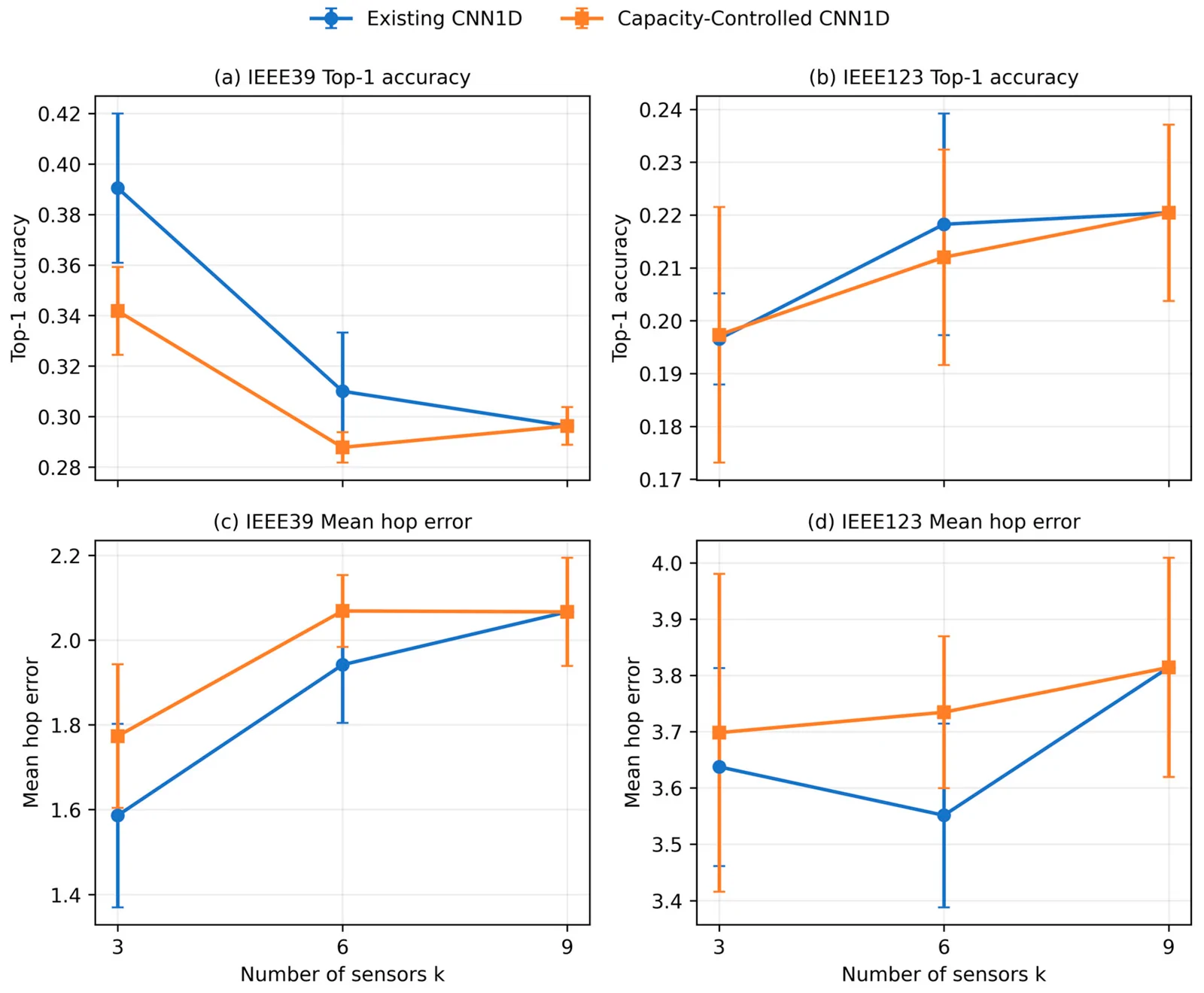
Sensor-budget comparison using identical outer condition folds, seeds 2026–2030, and five complete pooled-OOF estimates per curve. Panels show (**a**) IEEE39 Top-1, (**b**) IEEE123 Top-1, (**c**) IEEE39 mean hop, and (**d**) IEEE123 mean hop for the existing variable-input and 36-channel capacity-controlled CNN1D. Trainable parameter counts are reported in Table 8.

The original variable-input CNN1D remained non-monotonic across k = 3, 6, 9 (Figure 7). In the fixed-capacity 36-channel CNN1D, IEEE39 Top-1 was 0.342 ± 0.017, 0.288 ± 0.006, and 0.296 ± 0.007; additional sensors did not restore monotonicity. IEEE123 exact Top-1 increased modestly from 0.197 ± 0.024 to 0.212 ± 0.020 and 0.220 ± 0.017, whereas mean-hop error changed from 3.698 to 3.735 and 3.814 and therefore did not improve monotonically. Thus, the effect of increasing sensor count remains metric-dependent. Parameter-count confounding does not explain the IEEE39 curve; redundancy, representation, and optimization remain plausible contributors, and no causal optimal-budget claim is made.

**Table 8:** Fixed-capacity Top-1 results at k = 3, 6, 9. Values are means ± SDs across five pooled-OOF seeds.

System	k	Parameters	Top-1	Top-3	Macro-F1	Mean Hop
IEEE123	3	80,829	0.197 ± 0.024	0.469	0.175	3.698
IEEE123	6	80,829	0.212 ± 0.020	0.473	0.187	3.735
IEEE123	9	80,829	0.220 ± 0.017	0.477	0.195	3.814
IEEE39	3	54,153	0.342 ± 0.017	0.652	0.312	1.774
IEEE39	6	54,153	0.288 ± 0.006	0.603	0.247	2.069
IEEE39	9	54,153	0.296 ± 0.007	0.598	0.251	2.067

### 3.7. Classifier-Independence Evaluation

**Figure 8:**
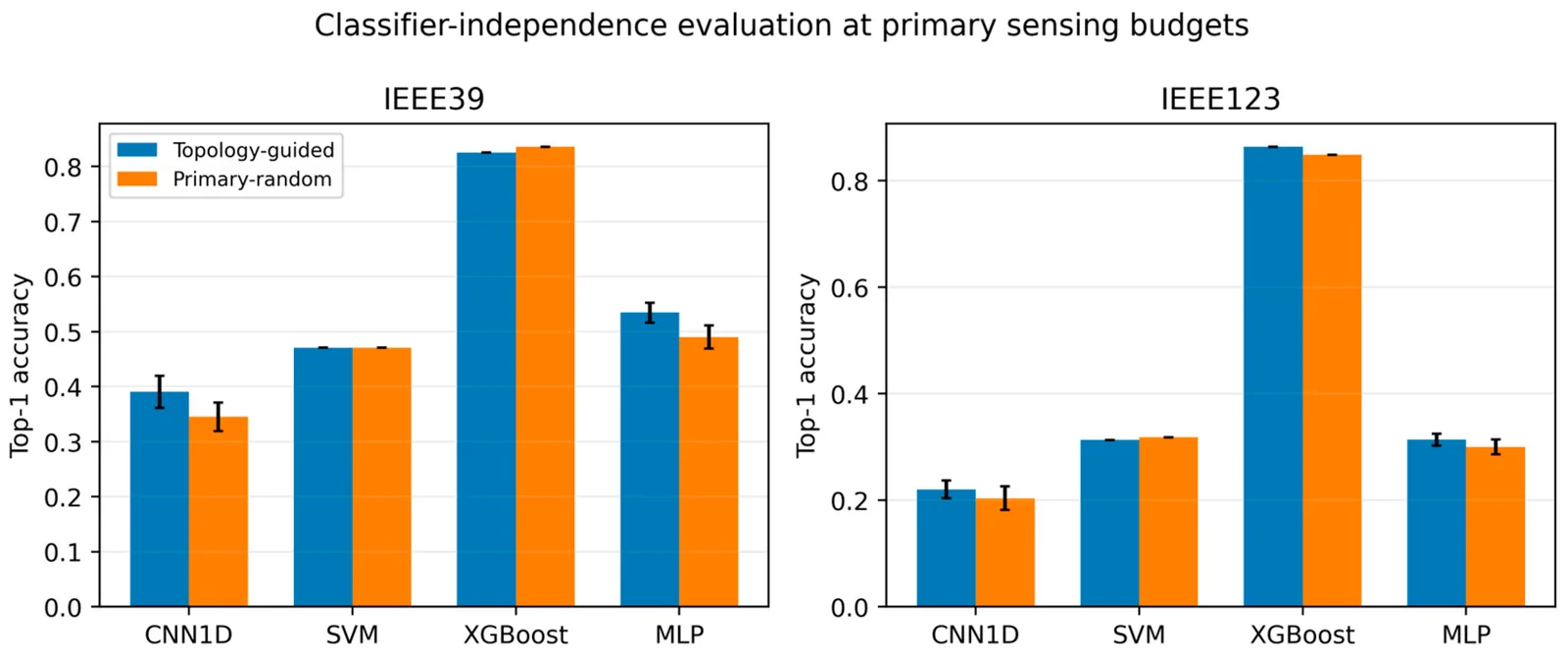
Classifier-independence evaluation at the primary sensing budgets. CNN1D and MLP error bars show the SD across five pooled-OOF seeds; deterministic SVM and XGBoost have one OOF estimate. Traditional classifiers use deterministic temporal summaries, whereas CNN1D uses the full time series.

At the primary budgets, topology-guided minus primary-random Top-1 differences were +0.0455/+0.0167 for CNN1D and +0.0444/+0.0136 for MLP for IEEE39/IEEE123. SVM was tied for IEEE39 and −0.0047 for IEEE123; XGBoost was −0.0106 for IEEE39 and +0.0148 for IEEE123. The direction is therefore not universal across classifiers. The placement algorithm remains classifier-independent in construction, but an empirical benefit must be reported per representation and classifier. XGBoost achieved the highest absolute Top-1 in both systems using summary features; this does not establish a strict algorithm ranking because its input representation differs from the CNN temporal tensor. The classifier-independence results are summarized in Figure 8 and Table 9.

### 3.8. Measurement-Uncertainty Sensitivity

**Figure 9:**
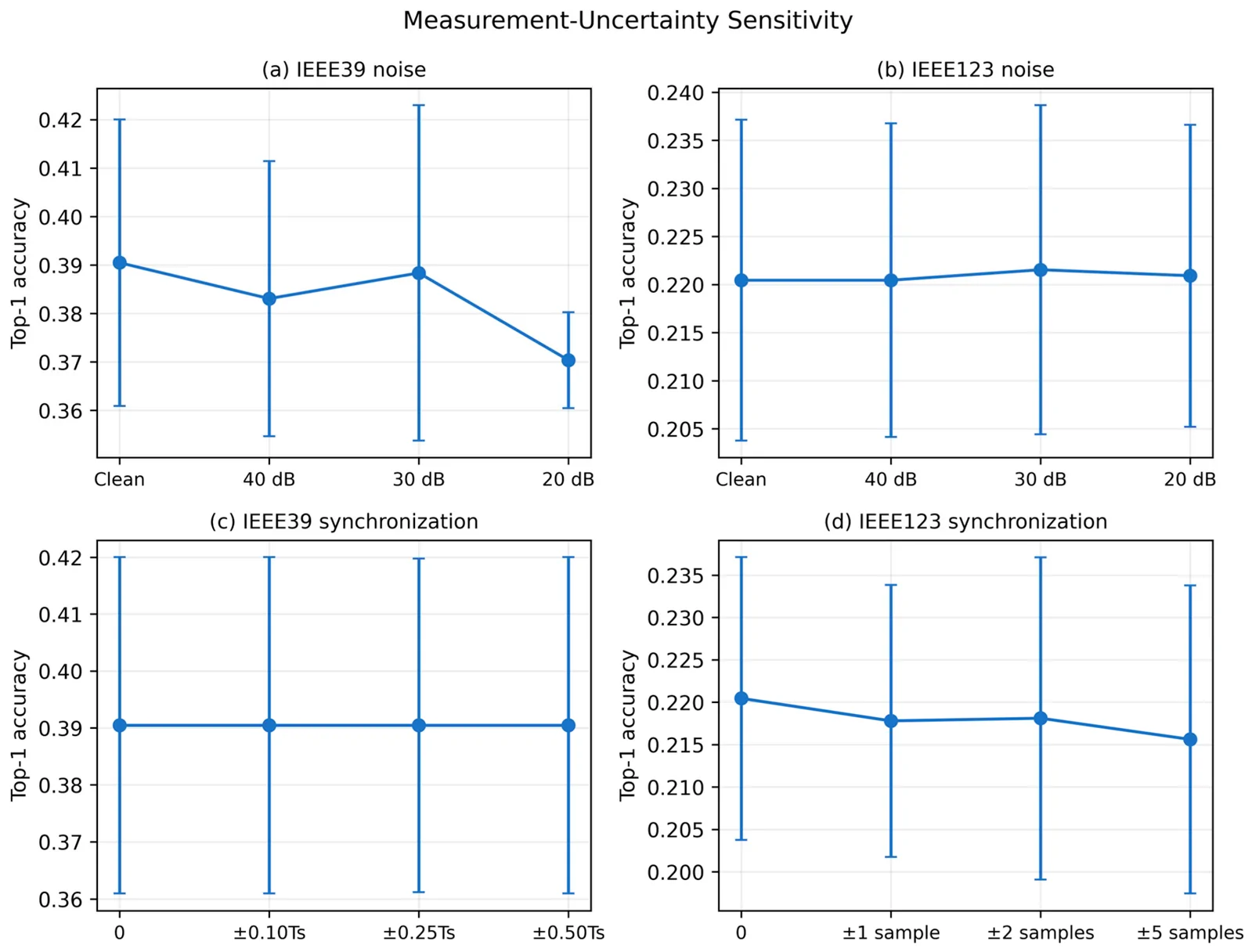
Measurement-uncertainty sensitivity: (**a**) IEEE39 noise, (**b**) IEEE123 noise, (**c**) IEEE39 synthetic fractional timing offsets on the native cycle-synchronous representation, and (**d**) IEEE123 integer sample shifts. Points and error bars are five-seed pooled-OOF means ± SDs.

The clean-to-20 dB Top-1 changed from 0.390 to 0.370 for IEEE39 and from 0.220 to 0.221 for IEEE123. At 20 dB, within-two-hop accuracy was 0.679 and 0.543, respectively. Timing effects were also limited: IEEE39 remained 0.390 through ±0.50Ts fractional offsets, while IEEE123 changed from 0.220 to 0.216 at ±5 samples. These are test-only perturbations of clean-trained models and do not establish hardware-noise immunity. Seed-level measurement-noise results are in Supplementary Table S1. The noise and timing sensitivity results are summarized in Figure 9.

### 3.9. Confusion and Topological Error Structure

**Figure 10:**
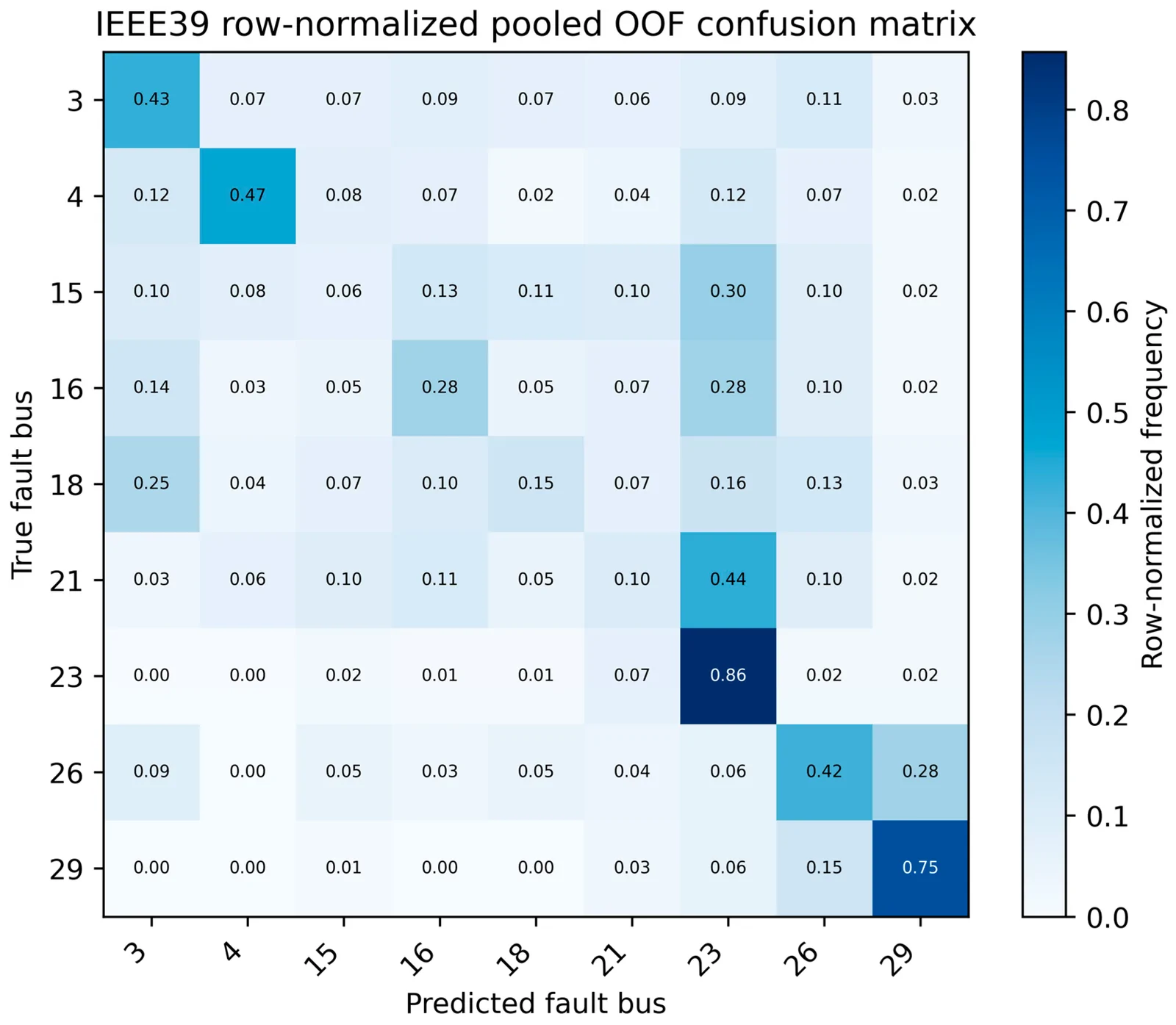
IEEE39 row-normalized 9 × 9 confusion matrix from complete pooled OOF predictions across all five CNN1D seeds at the primary topology-guided layout. Each row contains 21 events per seed, or 105 pooled predictions.

**Figure 11:**
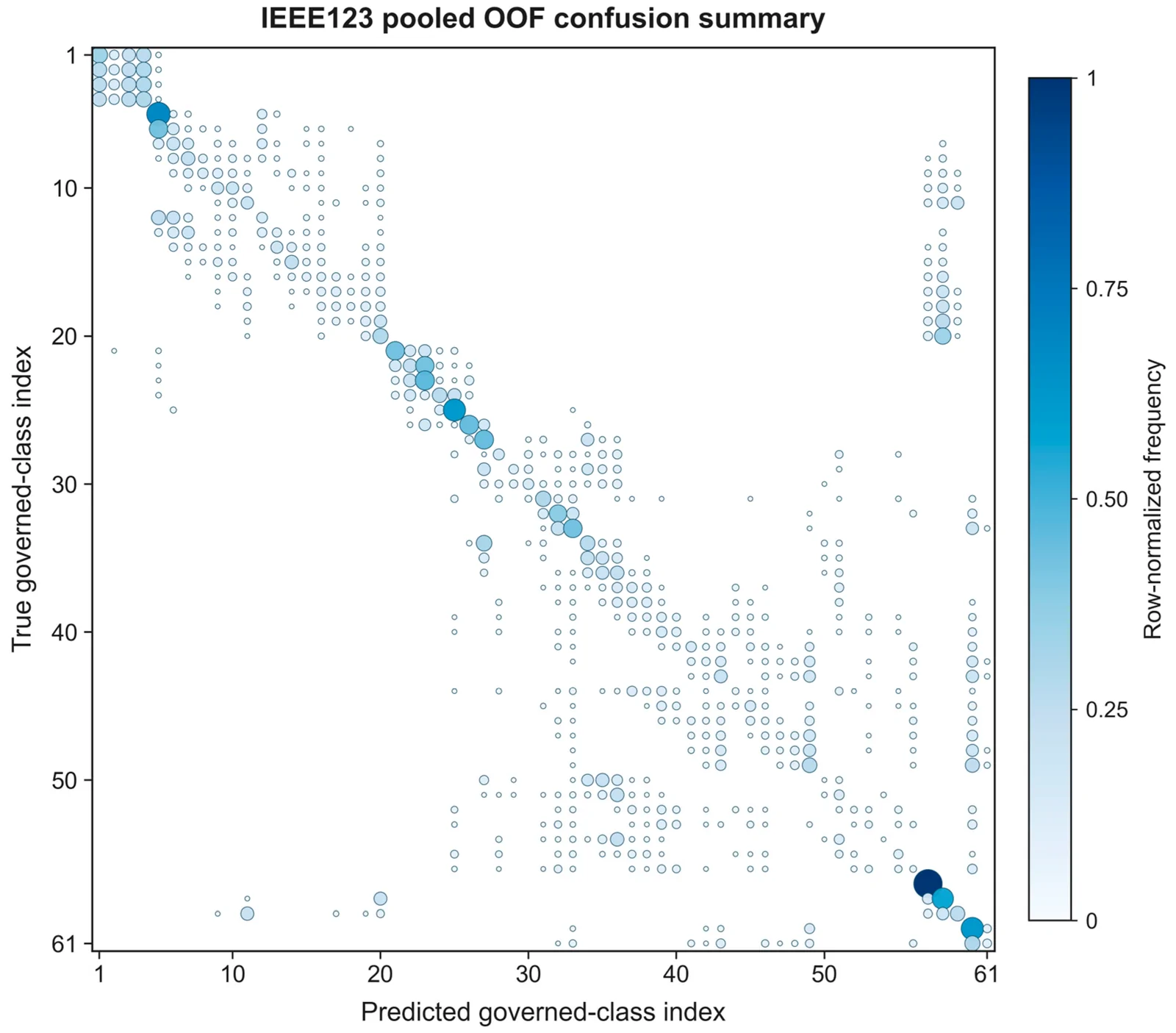
IEEE123 pooled five-seed row-normalized out-of-fold confusion summary under the topology-guided layout. Axes are indexed by the frozen governed-class order. Bubble area and color denote row-normalized frequency. To improve readability in the main text, all diagonal entries and off-diagonal entries with row-normalized frequency ≥0.02 are shown; the full dense confusion matrix is provided in Figure S1 of the Supplementary Materials.

**Figure 12:**
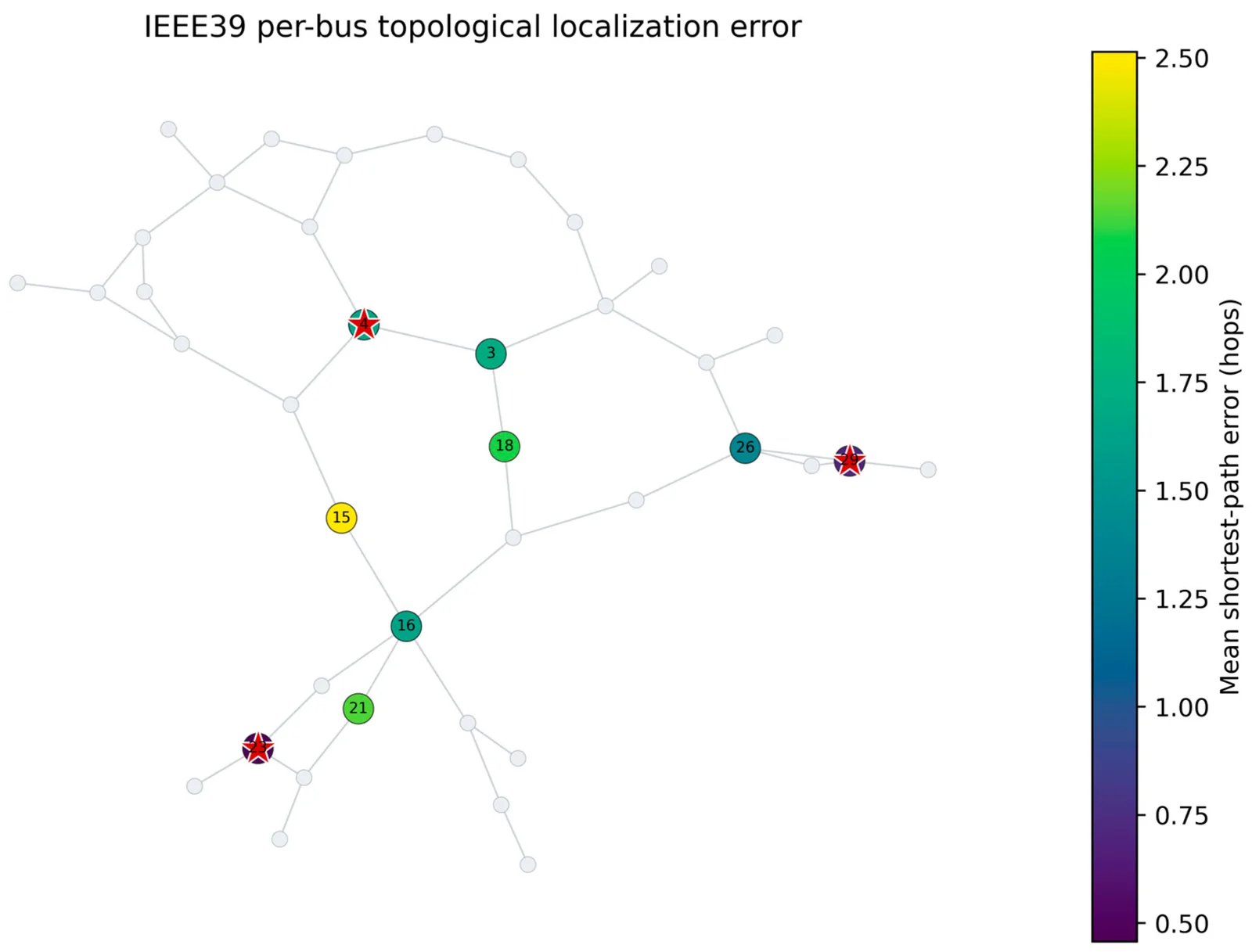
IEEE39 per-bus mean shortest-path localization error. Stars mark the primary k = 3 topology-guided measurement sensors; colored labeled nodes are the nine evaluated fault buses.

**Figure 13:**
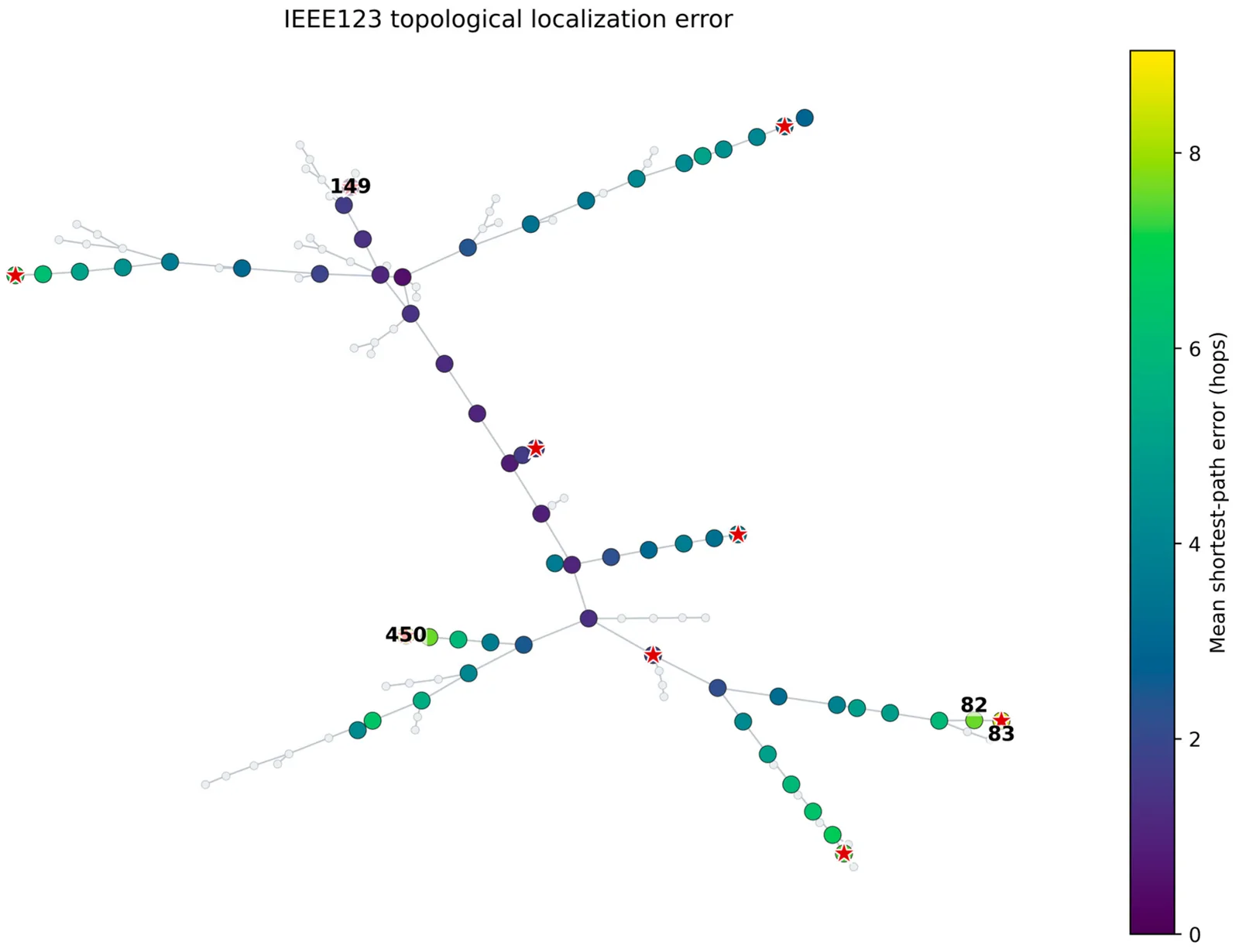
IEEE123 quotient-graph per-class mean shortest-path localization error. Colored nodes represent the 61 governed localization classes; selected labels are annotated for readability. Stars mark the nine primary topology-guided measurement sensors; gray nodes provide feeder connectivity context. The fully labeled map is provided in Supplementary Figure S2.

Five-seed pooled OOF confusion counts show strongly class-dependent behavior. IEEE39 buses 23 and 29 had Top-1 values of 0.857 and 0.752, whereas buses 15, 21, and 18 had the largest mean hop errors (2.514, 2.143, and 2.095). In IEEE123, class 149 was perfectly localized in the pooled predictions, but distal classes 450, 83, and 82 had mean hop errors of 9.048, 8.524, and 7.590. The diagonal and branch-structured confusion patterns show that errors are not uniformly distributed, yet substantial remote-error tails remain. The complete dense IEEE123 confusion matrix is provided in Figure S1 of the Supplementary Materials. The IEEE39 and IEEE123 confusion structures are shown in Figure 10 and Figure 11, respectively, and the corresponding topological error maps are shown in Figure 12 and Figure 13.

### 3.10. Fault-Type and Duration Shifts

**Figure 14:**
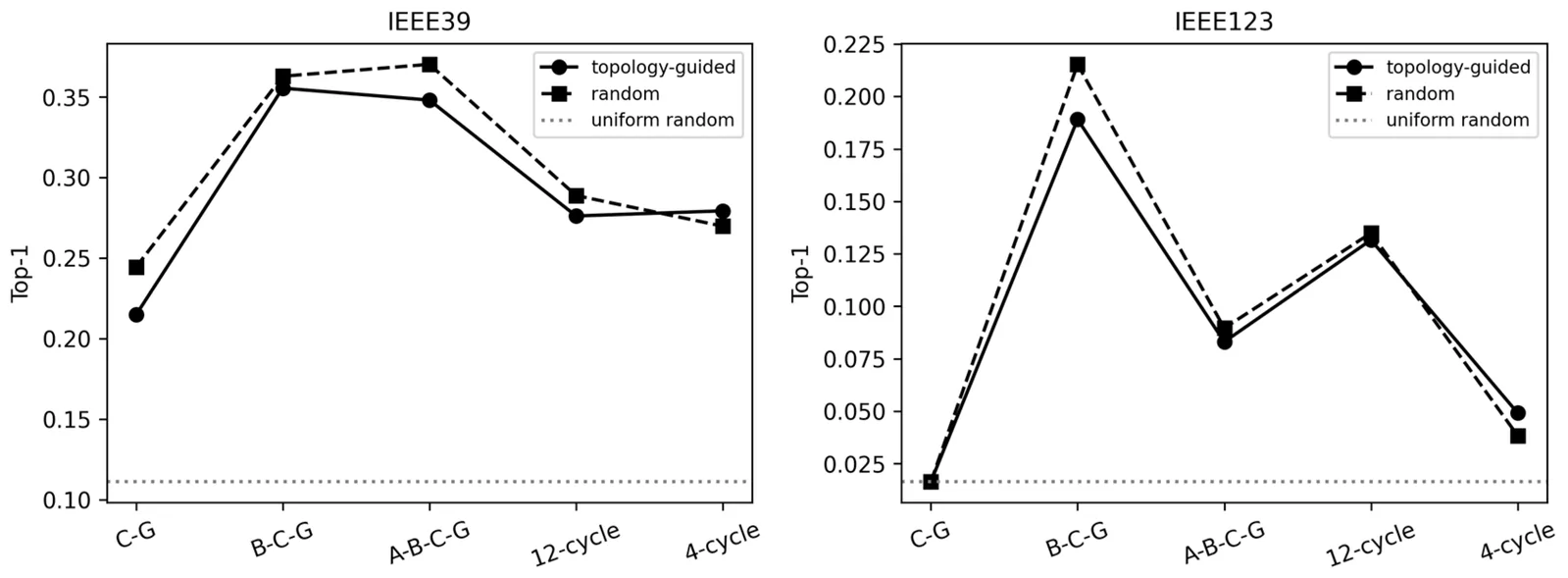
Primary localization results for complete fault-type holdouts (first three) and duration holdouts (last two). Lines show five-seed means; the dotted line is uniform-random Top-1.

Duration holdouts generally remain above uniform random in both systems, although they do not establish robustness to load, resistance, noise, or synchronization changes. Full unseen fault-type localization is harder. IEEE123 C–G Top-1 is exactly 1/61 for topology-guided placement, with no improvement over the uniform class reference. B–C–G and A–B–C–G holdouts remain above random, and the three IEEE39 type holdouts also remain above random. The fault-type and duration holdout results are compared in Figure 14.

Across the ten system-by-shift topology-versus-primary-random comparisons, only two Top-1 directions favor topology and neither has a significantly positive paired interval. Thus, the base-condition point benefit does not persist reliably under shift. The study does not support fault-type invariance, arbitrary-condition robustness, or universal transfer.

### 3.11. Extended Physical-Condition Evaluation

Across five complete pooled-OOF seeds, topology-guided placement achieved Top-1 0.3683, Top-3 0.6959, Macro-F1 0.3485, and mean-hop error 1.4665. Primary random achieved 0.3564, 0.7029, 0.3476, and 1.4903, respectively; distance-dispersion achieved 0.3222, 0.6335, 0.3010, and 1.9526. The median hop was 1.0 for topology and random and 1.6 for distance-dispersion. Table 10 reports the full seed-level summaries.

The topology-minus-random paired differences were +0.0119 for Top-1 (95% CI −0.0493 to 0.0898), +0.0009 for Macro-F1 (−0.0362 to 0.0367), and −0.0238 for mean-hop error (−0.2412 to 0.1505). Negative mean-hop differences favor topology. All three intervals include zero, so the extended analysis preserves the Top-1 and mean-hop point directions without establishing a statistically certain placement advantage. The corresponding paired bootstrap results are summarized in Table 11.

### 3.12. Load- and Resistance-Shift Evaluation

When trained only on L100, both layouts transferred poorly: topology/random Top-1 was 0.1220/0.1203 at L90 and 0.1280/0.1295 at L110. When trained only at 1 Ω, topology/random Top-1 was 0.4141/0.3891 at 0.1 Ω and 0.2776/0.2649 at 20 Ω. These one-factor tests address the requested IEEE39 operating-condition sensitivity, but the near-chance load-shift results and the degradation at high resistance preclude a broad robustness claim. The complete one-factor load- and resistance-shift results are summarized in Table 12.

## 4. Discussion

The topology-guided rule has a useful property: it can be executed before fault simulations or model training because it depends only on the operating graph and physically available sensors. The observed base-distribution point improvements are modest, and corrected uncertainty intervals cross zero against primary random. The value is therefore reproducibility and avoidance of demonstrably poor clustered layouts, not a claim of statistically certain dominance over any random draw.

The exhaustive IEEE39 layout-space result provides complementary evidence. Although the primary paired bus × condition confidence intervals against the prespecified random layout include zero, the topology-guided configuration ranked first among all 84 feasible k = 3 layouts for Top-1, Macro-F1, and mean-hop error under the frozen CNN1D protocol. The two findings are not contradictory; the bootstrap quantifies uncertainty across buses and factorial conditions, whereas the enumeration gives an exact rank within one finite candidate/layout/model setting. The latter therefore strengthens the reproducibility argument without establishing universal statistical or classifier-independent superiority.

Four evidence layers answer different questions. The original primary paired interval quantifies uncertainty for the prespecified topology-versus-random comparison under the 189-event base protocol and includes zero. The complete IEEE39 layout enumeration gives an exact rank within the finite IEEE39 k = 3 candidate space and places topology first for Top-1, Macro-F1, and mean hop. The reviewer-driven 1701-event extension tests a broader physical-condition grid; its Top-1 and mean-hop point directions favor topology, but all paired intervals include zero. The one-factor shift tests probe extrapolation rather than pooled-condition accuracy and reveal weak load transfer and resistance-dependent degradation. Together, these results strengthen finite-setting reproducibility while limiting statistical and operating-condition claims.

Topology and distance-dispersion are close because both favor spread. Their difference matters mainly in branched networks: full graph distance separates candidates on different branches even when their source distances coincide, whereas one-dimensional dispersion cannot. IEEE123’s radial structure can make the two objectives choose similar coverage, explaining their similar performance. IEEE39’s meshed structure provides more opportunities for full pairwise distance to change hop behavior. Neither method guarantees a globally optimal supervised layout.

IEEE123’s smaller point gain can arise from its 61-class output, heterogeneous feeder branches, and the limited ability of nine sensors to separate nearby quotient nodes. Its EMT signal has high temporal resolution, but more samples do not automatically solve spatial ambiguity. Conversely, IEEE39 has only nine location classes and three sensors among nine candidates. Absolute scores are not compared as domain difficulty because these factors are inseparable.

Exact Top-1 values of approximately 0.22 and 0.39 indicate that the present outputs are best interpreted as ranked localization evidence rather than stand-alone protection decisions. They can support inspection, restoration planning, or a second-stage search when Top-3 and hop errors are reported. A nearby incorrect label may still narrow the fault region, while a remote error carries a different operational cost. Cost-sensitive and uncertainty-aware regional evaluation is therefore a useful complement to exact-label accuracy.

Fixed-capacity results sharpen the budget interpretation. IEEE39 remained non-monotonic with constant trainable capacity, so first-layer parameter count is not a sufficient explanation; redundant channels, representation mismatch, and optimization remain plausible. IEEE123 showed a modest monotonic increase in exact Top-1, whereas mean-hop error did not improve monotonically. The systems therefore do not support a universal monotonic sensor-count law or a common optimal budget; the effect is metric-dependent.

The classifier comparison further shows that empirical placement benefit depends on the downstream representation and classifier. The topology direction was generally positive for CNN1D and MLP; SVM tied for IEEE39 and was slightly lower for topology for IEEE123; and XGBoost was lower for topology for IEEE39 and higher for IEEE123. The placement rule remains classifier-independent in construction, but its empirical benefit is classifier-/representation-dependent. In the present comparison, classifier/representation choice had a larger effect on absolute Top-1 accuracy than the difference between the two sensor layouts did. This does not diminish the placement problem, but it shows that layout effects must be interpreted jointly with the downstream representation and classifier. Because CNN1D uses the full temporal tensor whereas SVM, XGBoost, and MLP use deterministic summary features, these results are not a strict classifier leaderboard.

The IEEE123 C–G holdout produced Top-1 = 1/61. A plausible physical explanation is that phase-domain *V_a_*/*V_b_*/*V_c_* signatures are phase specific: withholding a complete single-phase-to-ground type removes that phase-specific distribution from training, and sparse spatial measurements can amplify the mismatch. The current representation does not explicitly expose positive-, negative-, or zero-sequence components. Symmetrical-component features, domain adaptation, few-shot adaptation, and physics-guided augmentation are therefore candidate extensions, but none of these were evaluated in the present study.

The two-system design improves methodological confidence without creating a transfer-learning experiment. IEEE39 projections and IEEE123 EMT waveforms have different semantics, sampling, and preprocessing. Models are trained and tested separately. What is shared is governance: frozen layouts, condition-level folds, complete OOF aggregation, and hop-aware evaluation. No IEEE39 model predicts IEEE123, and their absolute accuracies do not quantify the same physical challenge. The two benchmarks should therefore be interpreted as complementary validation scenarios rather than directly comparable levels of localization difficulty.

The validation remains simulation-based. IEEE39 now includes a matched 1701-event localization analysis at 90%, 100%, and 110% load and *R_pn_* = *R_ng_* = 0.1, 1, and 20 Ω, together with one-factor load and resistance shifts. The load-shift results are near chance and performance is lower at 20 Ω than at 0.1 Ω, so the study does not support robustness to arbitrary operating conditions. Matched non-nominal load/resistance localization remains unavailable for IEEE123 because referenced-model recompilation did not establish distinct stable L90/L110 waveforms. Converter-rich networks, missing data, parameter uncertainty, hardware-in-the-loop tests, and field measurements remain outside the evaluated scope.

Future work should establish a stable IEEE123 non-nominal operating-condition pipeline, evaluate matched load and resistance localization on that feeder, test sequence-domain representations for unseen phase faults, and examine converter-rich grids. Uncertainty-aware regional localization and robust sensor aggregation are complementary directions.

## 5. Conclusions

This study examined how sparse voltage placement and physically distinguishable labels shape fault-bus localization in independently trained IEEE39 and IEEE123 simulations. In the original base protocols, topology-guided Top-1 point differences over primary random were positive, but their paired intervals included zero. Under the frozen IEEE39 CNN1D protocol, the topology-guided layout nevertheless ranked first among all 84 feasible k = 3 layouts for Top-1, Macro-F1, and mean-hop error. In the reviewer-driven 1701-event extension, topology again had a positive Top-1 point difference (+0.0119) and lower mean-hop error (−0.0238), but all extended paired intervals included zero.

Load-shift Top-1 remained near chance, and resistance-shift performance degraded at 20 Ω. These findings bound the evidence to the tested IEEE39 conditions and leave matched IEEE123 non-nominal evaluation unresolved. The fixed-capacity ablation remained non-monotonic for IEEE39, classifier benefit was mixed, and the unseen IEEE123 C–G result remained at the uniform reference. The method supports reproducible offline measurement planning and ranked regional assessment, but it does not establish universal superiority or replace field and hardware validation.

## Figures and Tables

**Table 1:** Frozen physical and dataset definitions.

System	Domain	Sampling	Raw Signal Semantics	Locations	Events	Candidate Sensors	Primary k
IEEE39	Cycle-synchronous electromechanical domain	1/60 s	Signed *V_ab_*/*V_bc_*/*V_ca_* projection; unit V	9	189	9	3
IEEE123	Electromagnetic-transient domain	20 kHz	Physical phase-to-ground *V_ab_*/*V_bc_*/*V_ca_*; unit V	61	1281	59	9

**Table 2:** Frozen placement controls recovered from the executed layout-generation code.

Method	Geometry/Input	Selection Objective	Tie Rule	Randomness
Topology-guided	Full pairwise quotient/native graph distance	Greedy maximum minimum graph distance	Frozen candidate order	None
Distance-dispersion	One-dimensional source-distance coordinate	Maximum minimum absolute coordinate separation	Frozen order	None
Degree-centrality	Unweighted node degree	Descending degree	Frozen order	None
Primary random	Frozen eligible candidate pool	Uniform sampling without replacement	Not applicable	Frozen seed

**Table 3:** CNN1D architecture recovered from the executed training code.

Layer	Input Shape	Output Shape	Kernel Size	Stride	Activation	Dropout	Parameters
Input	4 k × T	4 k × T	-	-	-	-	0
Conv1	4 k × T	64 × T/2	9	2	ReLU	0.1	2304 k + 64
Conv2	64 × T/2	64 × T/4	7	2	ReLU	0	28,736
Adaptive AvgPool	64 × T/4	64 × 8	-	-	-	0	0
Linear	512	C	-	-	softmax at evaluation	0	512 C + C

Note: T is the input sequence length, k is the number of sensors, and C is the number of localization classes (C = 9 for IEEE39 and C = 61 for IEEE123).

**Table 4:** Auxiliary fault-type results for the primary topology-guided layouts. The mean and SD are across five complete pooled-OOF seeds; duration columns report the frozen duration holdouts. IEEE39 and IEEE123 are trained separately. The uniform seven-class random reference is 1/7 = 0.1429.

System	Base Accuracy	Base Macro-F1	Duration Accuracy Range	Duration Macro-F1 Range	Random Reference
IEEE123	0.9961	0.9961	0.8384–1.0000	0.8286–1.0000	0.1429
IEEE39	0.7566	0.7561	0.5714–0.8254	0.5595–0.8162	0.1429

Note: Across the five pooled-OOF model seeds, the standard deviations of the two base metrics were 0.0000 for both systems.

**Table 5:** Main topology-guided CNN1D results. The mean and SD are across five complete pooled-OOF seeds; systems are trained separately.

System	Seeds	Top-1	Top-1 SD	Top-3	Macro-F1	Mean Hop	Median Hop	≤1 Hop	≤2 Hops
IEEE123	5	0.220	0.017	0.477	0.195	3.814	2.000	0.429	0.546
IEEE39	5	0.390	0.030	0.686	0.358	1.586	1.200	0.534	0.685

**Table 6:** Absolute placement results. The mean and SD are across five complete pooled-OOF seeds. “Primary random” denotes the protocol-frozen random layout specified before model evaluation, not the best random draw.

System	Method	Top-1	Top-1 SD	Macro-F1	Macro-F1 SD	Mean Hop	Hop SD
IEEE123	Degree-centrality	0.198	0.016	0.173	0.015	4.293	0.133
IEEE123	Distance-dispersion	0.218	0.012	0.199	0.014	4.012	0.112
IEEE123	Primary random	0.204	0.023	0.175	0.021	3.901	0.238
IEEE123	Topology-guided	0.220	0.017	0.195	0.015	3.814	0.195
IEEE39	Degree-centrality	0.273	0.017	0.238	0.021	2.267	0.169
IEEE39	Distance-dispersion	0.324	0.022	0.298	0.024	2.049	0.153
IEEE39	Topology-guided	0.390	0.030	0.358	0.038	1.586	0.216
IEEE39	Primary random	0.345	0.026	0.306	0.030	1.726	0.193

**Table 7:** Primary paired bus × condition Cartesian-product bootstrap (1000 replicates). Difference is topology-guided minus primary random; the interval is a 95% confidence interval, and a negative mean-hop difference is favorable. Full sensitivity analyses are in Supplementary Table S3.

System	Comparison	Metric	Paired Difference	95% CI Lower	95% CI Upper	CI Excludes Zero
IEEE123	Topology-guided vs. primary random	Top-1 accuracy	0.0167	−0.0180	0.0567	False
IEEE123	Topology-guided vs. primary random	Macro-F1	0.0182	−0.0070	0.0337	False
IEEE123	Topology-guided vs. primary random	Mean hop error	−0.0873	−0.4287	0.2523	False
IEEE39	Topology-guided vs. primary random	Top-1 accuracy	0.0455	−0.0296	0.1397	False
IEEE39	Topology-guided vs. primary random	Macro-F1	0.0508	−0.0205	0.0759	False
IEEE39	Topology-guided vs. primary random	Mean hop error	−0.1397	−0.4317	0.0933	False

**Table 9:** Classifier-independence Top-1 accuracy at the primary sensing budgets. SD is not reported for deterministic classifiers with a single OOF estimate.

System	Classifier	Placement	n	Top-1	SD	≤1 Hop	Mean Hop
IEEE123	CNN1D	Primary random	5	0.204	0.023	0.419	3.901
IEEE123	CNN1D	Topology-guided	5	0.220	0.017	0.429	3.814
IEEE123	MLP	Primary random	5	0.300	0.014	0.577	2.541
IEEE123	MLP	Topology-guided	5	0.314	0.011	0.588	2.294
IEEE123	SVM	Primary random	1	0.318	—	0.598	2.237
IEEE123	SVM	Topology-guided	1	0.313	—	0.585	2.240
IEEE123	XGBoost	Primary random	1	0.849	—	0.891	1.184
IEEE123	XGBoost	Topology-guided	1	0.863	—	0.904	1.087
IEEE39	CNN1D	Primary random	5	0.345	0.026	0.508	1.726
IEEE39	CNN1D	Topology-guided	5	0.390	0.030	0.534	1.586
IEEE39	MLP	Primary random	5	0.490	0.021	0.703	1.004
IEEE39	MLP	Topology-guided	5	0.534	0.018	0.684	0.984
IEEE39	SVM	Primary random	1	0.471	—	0.608	1.265
IEEE39	SVM	Topology-guided	1	0.471	—	0.603	1.275
IEEE39	XGBoost	Primary random	1	0.836	—	0.910	0.296
IEEE39	XGBoost	Topology-guided	1	0.825	—	0.905	0.339

**Table 10:** Reviewer-driven IEEE39 extended-condition localization. Values are means ± SDs across five complete pooled-OOF seeds.

Placement	Top-1	Top-3	Macro-F1	Mean Hop	≤1 Hop	≤2 Hops
Topology-guided	0.3683 ± 0.0103	0.6959 ± 0.0130	0.3485 ± 0.0123	1.4665 ± 0.0502	0.5593 ± 0.0207	0.7367 ± 0.0180
Primary random	0.3564 ± 0.0100	0.7029 ± 0.0263	0.3476 ± 0.0140	1.4903 ± 0.0788	0.5505 ± 0.0197	0.7529 ± 0.0321
Distance-dispersion	0.3222 ± 0.0169	0.6335 ± 0.0244	0.3010 ± 0.0181	1.9526 ± 0.0496	0.4976 ± 0.0180	0.6061 ± 0.0183

**Table 11:** Extended paired bus × condition Cartesian-product bootstrap. Difference is topology-guided minus primary random; a negative mean-hop difference is favorable.

Metric	Difference	95% CI Lower	95% CI Upper	Direction Favoring Topology	CI Excludes Zero	Replicates
Top-1	+0.0119	−0.0493	0.0898	Positive	No	5000
Macro-F1	+0.0009	−0.0362	0.0367	Positive	No	5000
Mean hop	−0.0238	−0.2412	0.1505	Negative	No	5000

**Table 12:** One-factor load- and resistance-shift evaluation. Values are five-seed means; test-factor samples were excluded from all train-only operations.

Training	Test	Placement	Top-1	Top-3	Macro-F1	Mean Hop	≤1/≤2 Hop
L100	L90	Topology-guided	0.1220	0.3693	0.0604	2.5570	0.3337/0.3869
L100	L90	Primary random	0.1203	0.3601	0.0514	2.5295	0.3259/0.3891
L100	L110	Topology-guided	0.1280	0.3616	0.0583	2.1672	0.3446/0.5330
L100	L110	Primary random	0.1295	0.3584	0.0624	2.1175	0.3485/0.5534
R = 1 Ω	R = 0.1 Ω	Topology-guided	0.4141	0.7407	0.3959	1.3231	0.5996/0.7686
R = 1 Ω	R = 0.1 Ω	Primary random	0.3891	0.7305	0.3760	1.3732	0.5834/0.7901
R = 1 Ω	R = 20 Ω	Topology-guided	0.2776	0.5806	0.2671	1.7072	0.5026/0.6751
R = 1 Ω	R = 20 Ω	Primary random	0.2649	0.5683	0.2544	1.8000	0.4649/0.6829

## Data Availability

The processed data supporting this study, including derived split manifests, quotient mappings, frozen layouts, prediction tables, aggregate results, analysis scripts, minimal schemas, and hashes, are available from the corresponding author upon reasonable request. Raw MATLAB files and upstream model assets are not publicly deposited because of file size and third-party model licensing. No public repository URL or DOI is claimed.

## Supplementary Materials

The following supporting information can be downloaded at: https://www.mdpi.com/article/10.3390/s26196203/s1. Table S1: Measurement-noise sensitivity results; Table S2: Fault-type and duration-shift results; Table S3: Bootstrap sensitivity analyses; Table S4: Frozen IEEE39 extended physical-condition configuration; Table S5: Extended pooled-OOF seed metrics; Table S6: Topology-guided minus primary-random paired bootstrap; Table S7: Load- and resistance-shift evaluation; Table S8: IEEE39 complete 84-layout analysis; Table S9: Structural layout metrics; Figure S1: Full IEEE123 row-normalized pooled out-of-fold confusion matrix under the topology-guided layout; Figure S2: Full IEEE123 quotient-graph per-class mean-hop error map under the topology-guided layout; Figure S3: IEEE39 extended-condition pooled-OOF evaluation; Figure S4: Load shift after training on L100; Figure S5: Resistance shift after training at *R_pn_* = *R_ng_* = 1 Ω; Figure S6: Complete IEEE39 84-layout CNN1D performance landscape under the frozen base protocol; Figure S7: IEEE39 topology-only structural metric distributions across all 84 layouts; Figure S8: IEEE123 topology-only structural metric distributions for 10,000 frozen random layouts and four named layouts.
