# Supplementary material for: Topology-Guided Sparse Voltage Measurement Placement for Transient-Voltage Fault-Bus Localization: A Physically Governed Dual-System Evaluation
Source: Sensors (Basel). 2026 Sep 30;26(19):6203. doi: 10.3390/s26196203 (PMC13667557; doi:10.3390/s26196203)
Supplement: Supplementary file 1 [file sensors-26-06203-s001.zip › sensors-4598232-supplementary.pdf]

Supplementary Materials for Manuscript sensors-4598232

These Supplementary Materials provide dataset definitions, layout manifests, seed-level results, robustness analyses, distribution-shift evaluations, complete layout-space results, and structural localization evidence supporting the main manuscript. All reported values trace to frozen executed outputs.

S1 System and Dataset Definitions

Frozen IEEE39 and IEEE123 signal domains, event definitions, class counts, candidate sets, and primary sensing budgets.

S2 Frozen Measurement Candidates and Layouts

Candidate-order, deterministic-layout, and random-layout manifests.

S3 IEEE123 Quotient Graph Mapping

Canonical representatives, excluded labels, five frozen contractions, quotient edges, and raw-to-governed label mapping.

S4 Condition Level Fold Manifests

Outer and inner condition-fold definitions, seed policy, and leakage checks.

S5 Additional Base Protocol Metrics

Complete pooled-OOF Top-k, Macro-F1, and graph-hop metrics supporting the main tables.

S6 Fixed Capacity Ablation

Completed 36-channel fixed-capacity k=3,6,9 results and parameter-count audit.

S7 Multi Classifier Comparison

Completed CNN1D, SVM, XGBoost, and MLP comparison under identical frozen layouts and folds.

S8 Measurement Noise Sensitivity

Completed clean, 40, 30, and 20 dB test-only Gaussian-noise evaluation.

Table S1. Measurement-noise sensitivity results, classification metrics. Values are rounded to four decimals from the frozen noise_robustness.csv output.

System	Placement	k	Seed	Test noise	Top-1	Top-3	Macro-F1
IEEE39	Topology-guided	3	2026	clean	0.4021	0.6825	0.3748
IEEE39	Topology-guided	3	2026	40 dB	0.3968	0.6878	0.3681
IEEE39	Topology-guided	3	2026	30 dB	0.4127	0.6931	0.3846
IEEE39	Topology-guided	3	2026	20 dB	0.3810	0.6931	0.3394
IEEE39	Topology-guided	3	2027	clean	0.4127	0.6931	0.3858
IEEE39	Topology-guided	3	2027	40 dB	0.4021	0.6878	0.3780
IEEE39	Topology-guided	3	2027	30 dB	0.4074	0.6931	0.3797
IEEE39	Topology-guided	3	2027	20 dB	0.3757	0.6931	0.3481
IEEE39	Topology-guided	3	2028	clean	0.4021	0.6931	0.3655
IEEE39	Topology-guided	3	2028	40 dB	0.3968	0.6931	0.3600
IEEE39	Topology-guided	3	2028	30 dB	0.4021	0.6825	0.3651
IEEE39	Topology-guided	3	2028	20 dB	0.3757	0.6720	0.3433

System	Placement	k	Seed	Test noise	Top-1	Top-3	Macro-F1
IEEE39	Topology-guided	3	2029	clean	0.3386	0.6614	0.2913
IEEE39	Topology-guided	3	2029	40 dB	0.3333	0.6667	0.2836
IEEE39	Topology-guided	3	2029	30 dB	0.3280	0.6455	0.2743
IEEE39	Topology-guided	3	2029	20 dB	0.3598	0.6455	0.3232
IEEE39	Topology-guided	3	2030	clean	0.3968	0.6984	0.3720
IEEE39	Topology-guided	3	2030	40 dB	0.3862	0.6984	0.3622
IEEE39	Topology-guided	3	2030	30 dB	0.3915	0.6984	0.3667
IEEE39	Topology-guided	3	2030	20 dB	0.3598	0.6772	0.3378
IEEE123	Topology-guided	9	2026	clean	0.2467	0.5105	0.2179
IEEE123	Topology-guided	9	2026	40 dB	0.2459	0.5105	0.2172
IEEE123	Topology-guided	9	2026	30 dB	0.2482	0.5121	0.2192
IEEE123	Topology-guided	9	2026	20 dB	0.2436	0.5152	0.2140
IEEE123	Topology-guided	9	2027	clean	0.2108	0.4793	0.1855
IEEE123	Topology-guided	9	2027	40 dB	0.2108	0.4793	0.1856
IEEE123	Topology-guided	9	2027	30 dB	0.2108	0.4778	0.1855
IEEE123	Topology-guided	9	2027	20 dB	0.2092	0.4801	0.1855
IEEE123	Topology-guided	9	2028	clean	0.2264	0.4668	0.2023
IEEE123	Topology-guided	9	2028	40 dB	0.2264	0.4684	0.2021
IEEE123	Topology-guided	9	2028	30 dB	0.2279	0.4645	0.2033
IEEE123	Topology-guided	9	2028	20 dB	0.2303	0.4668	0.2058
IEEE123	Topology-guided	9	2029	clean	0.2139	0.4676	0.1900
IEEE123	Topology-guided	9	2029	40 dB	0.2147	0.4668	0.1906
IEEE123	Topology-guided	9	2029	30 dB	0.2155	0.4676	0.1920
IEEE123	Topology-guided	9	2029	20 dB	0.2155	0.4715	0.1912
IEEE123	Topology-guided	9	2030	clean	0.2045	0.4621	0.1790
IEEE123	Topology-guided	9	2030	40 dB	0.2045	0.4621	0.1790
IEEE123	Topology-guided	9	2030	30 dB	0.2053	0.4637	0.1797
IEEE123	Topology-guided	9	2030	20 dB	0.2061	0.4660	0.1802

Table S1 (continued). Measurement-noise sensitivity results, graph-error metrics from the same frozen output.

System	Placement	Seed	Test noise	Mean hop	Median hop	Within 1	Within 2
IEEE39	Topology-guided	2026	clean	1.5820	1.0000	0.5291	0.6772
IEEE39	Topology-guided	2026	40 dB	1.5979	1.0000	0.5185	0.6772
IEEE39	Topology-guided	2026	30 dB	1.5608	1.0000	0.5397	0.6878
IEEE39	Topology-guided	2026	20 dB	1.6825	1.0000	0.5026	0.6455
IEEE39	Topology-guided	2027	clean	1.4497	1.0000	0.5661	0.7143
IEEE39	Topology-guided	2027	40 dB	1.4868	1.0000	0.5556	0.7090
IEEE39	Topology-guided	2027	30 dB	1.4709	1.0000	0.5661	0.7090
IEEE39	Topology-guided	2027	20 dB	1.4868	1.0000	0.5397	0.7196
IEEE39	Topology-guided	2028	clean	1.5397	1.0000	0.5450	0.7196
IEEE39	Topology-guided	2028	40 dB	1.5608	1.0000	0.5397	0.7143
IEEE39	Topology-guided	2028	30 dB	1.5661	1.0000	0.5450	0.7037
IEEE39	Topology-guided	2028	20 dB	1.5608	1.0000	0.5503	0.7196
IEEE39	Topology-guided	2029	clean	1.9524	2.0000	0.4550	0.5820
IEEE39	Topology-guided	2029	40 dB	1.9630	2.0000	0.4550	0.5767
IEEE39	Topology-guided	2029	30 dB	2.0053	2.0000	0.4444	0.5714
IEEE39	Topology-guided	2029	20 dB	1.8201	2.0000	0.4762	0.6138
IEEE39	Topology-guided	2030	clean	1.4074	1.0000	0.5767	0.7302
IEEE39	Topology-guided	2030	40 dB	1.4603	1.0000	0.5608	0.7196

System	Placement	Seed	Test noise	Mean hop	Median hop	Within 1	Within 2
IEEE39	Topology-guided	2030	30 dB	1.4286	1.0000	0.5661	0.7249
IEEE39	Topology-guided	2030	20 dB	1.5873	1.0000	0.5291	0.6984
IEEE123	Topology-guided	2026	clean	3.5980	2.0000	0.4676	0.5738
IEEE123	Topology-guided	2026	40 dB	3.6003	2.0000	0.4676	0.5738
IEEE123	Topology-guided	2026	30 dB	3.5917	2.0000	0.4699	0.5753
IEEE123	Topology-guided	2026	20 dB	3.6105	2.0000	0.4676	0.5722
IEEE123	Topology-guided	2027	clean	4.0546	2.0000	0.4052	0.5168
IEEE123	Topology-guided	2027	40 dB	4.0578	2.0000	0.4059	0.5160
IEEE123	Topology-guided	2027	30 dB	4.0562	2.0000	0.4028	0.5160
IEEE123	Topology-guided	2027	20 dB	4.0734	2.0000	0.4028	0.5144
IEEE123	Topology-guided	2028	clean	3.8415	2.0000	0.4379	0.5464
IEEE123	Topology-guided	2028	40 dB	3.8392	2.0000	0.4379	0.5457
IEEE123	Topology-guided	2028	30 dB	3.8462	2.0000	0.4372	0.5464
IEEE123	Topology-guided	2028	20 dB	3.8423	2.0000	0.4403	0.5441
IEEE123	Topology-guided	2029	clean	3.6378	2.0000	0.4340	0.5699
IEEE123	Topology-guided	2029	40 dB	3.6378	2.0000	0.4333	0.5699
IEEE123	Topology-guided	2029	30 dB	3.6386	2.0000	0.4333	0.5691
IEEE123	Topology-guided	2029	20 dB	3.6635	2.0000	0.4325	0.5675
IEEE123	Topology-guided	2030	clean	3.9391	2.0000	0.3989	0.5215
IEEE123	Topology-guided	2030	40 dB	3.9422	2.0000	0.3989	0.5222
IEEE123	Topology-guided	2030	30 dB	3.9235	2.0000	0.4012	0.5254
IEEE123	Topology-guided	2030	20 dB	3.9594	2.0000	0.3981	0.5191

S9 Synchronization Sensitivity

Completed system-specific timing-perturbation evaluation.

S10 Full IEEE123 Confusion Matrix

Supplementary Figure S1 is the complete 61-class row-normalized pooled OOF confusion matrix. The underlying raw counts are available from the corresponding author together with the processed analysis data.

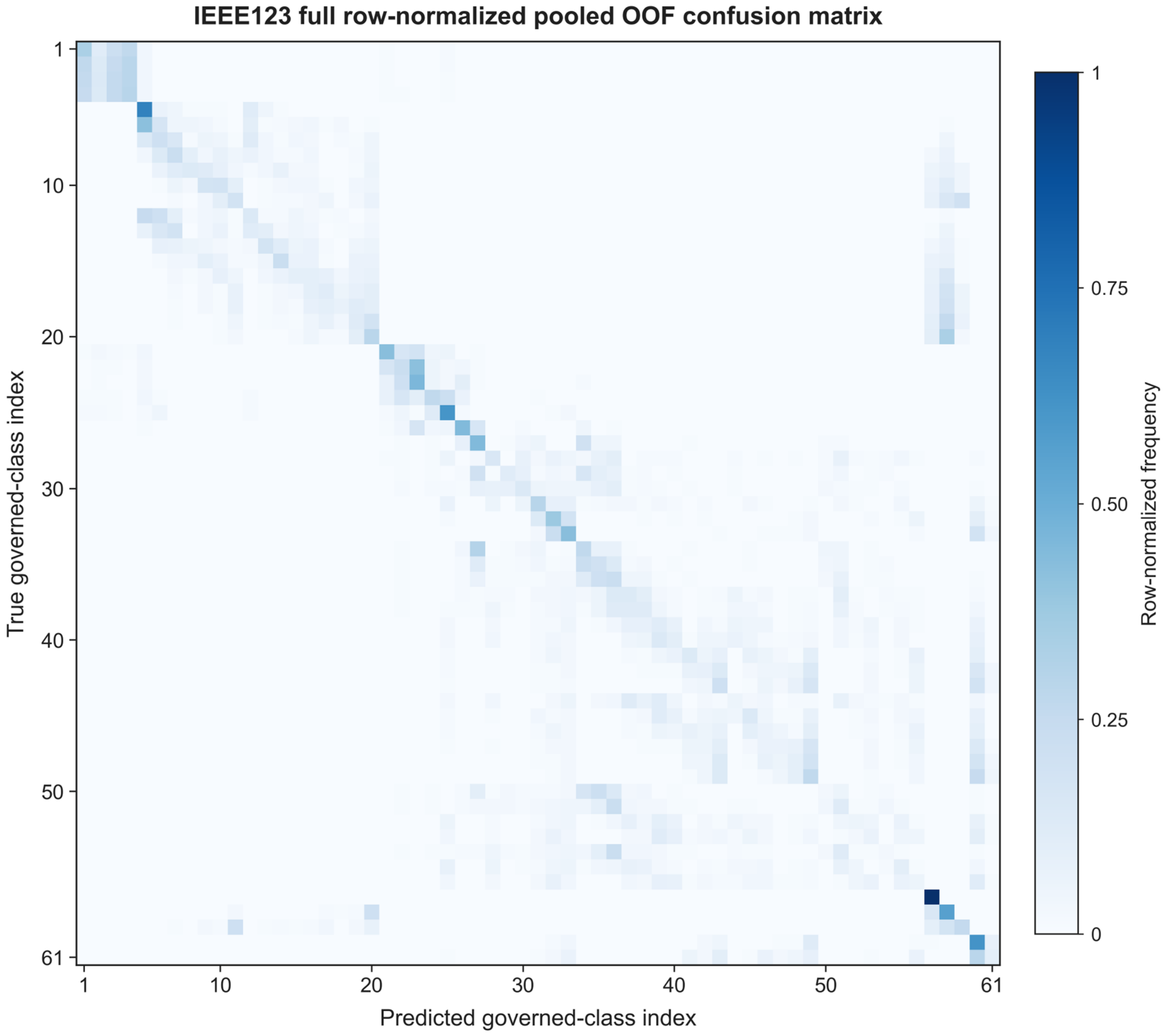

Figure S1. Full IEEE123 row-normalized pooled out-of-fold confusion matrix under the topology-guided layout, shown in the frozen governed-class order. Both axes use governed-class indices rather than full bus labels to maintain readability. This figure provides the complete dense matrix corresponding to the summarized main-text visualization in Figure 11.

S11 Full IEEE123 Topological Error Map

Supplementary Figure S2 provides the complete labeled IEEE123 quotient-graph error map used by the main-text summary.

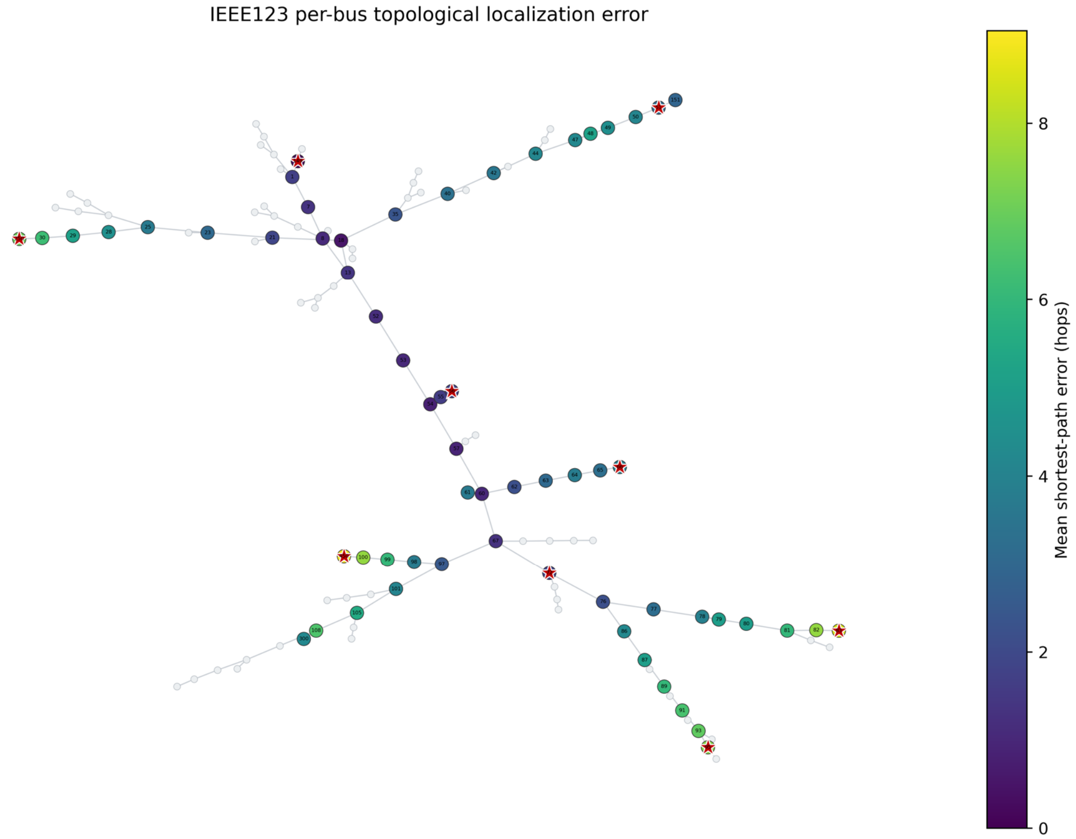

Figure S2. Full IEEE123 quotient-graph per-class mean-hop error map under the topology-guided layout.

S12 Fault Type and Duration Shift Results

Completed frozen fault-type and duration holdouts, including the explicitly bounded near-random IEEE123 C-G result.

Table S2. Fault-type and duration-shift results:auxiliary fault-type-identification duration holdouts. Shift-D tests 12-cycle events after training on 4- and 6-cycle events; Shift-E tests 4-cycle events after training on 6- and 12-cycle events. Values are rounded to four decimals from the frozen phase25_stage1_shift_metrics.csv output.

System	Placement	Holdout condition	Seed	Events	Accuracy	Macro-F1
IEEE123	topology-guided	12-cycle duration	2026	427	0.8384	0.8286
IEEE123	topology-guided	12-cycle duration	2027	427	0.8384	0.8286
IEEE123	topology-guided	12-cycle duration	2028	427	0.8384	0.8286
IEEE123	topology-guided	12-cycle duration	2029	427	0.8384	0.8286
IEEE123	topology-guided	12-cycle duration	2030	427	0.8384	0.8286
IEEE123	topology-guided	4-cycle duration	2026	427	1.0000	1.0000
IEEE123	topology-guided	4-cycle duration	2027	427	1.0000	1.0000
IEEE123	topology-guided	4-cycle duration	2028	427	1.0000	1.0000
IEEE123	topology-guided	4-cycle duration	2029	427	1.0000	1.0000
IEEE123	topology-guided	4-cycle duration	2030	427	1.0000	1.0000
IEEE123	random	12-cycle duration	2026	427	1.0000	1.0000

System	Placement	Holdout condition	Seed	Events	Accuracy	Macro-F1
IEEE123	random	12-cycle duration	2027	427	1.0000	1.0000
IEEE123	random	12-cycle duration	2028	427	1.0000	1.0000
IEEE123	random	12-cycle duration	2029	427	1.0000	1.0000
IEEE123	random	12-cycle duration	2030	427	1.0000	1.0000
IEEE123	random	4-cycle duration	2026	427	0.9883	0.9883
IEEE123	random	4-cycle duration	2027	427	0.9883	0.9883
IEEE123	random	4-cycle duration	2028	427	0.9883	0.9883
IEEE123	random	4-cycle duration	2029	427	0.9883	0.9883
IEEE123	random	4-cycle duration	2030	427	0.9883	0.9883
IEEE39	topology-guided	12-cycle duration	2026	63	0.5714	0.5595
IEEE39	topology-guided	12-cycle duration	2027	63	0.5714	0.5595
IEEE39	topology-guided	12-cycle duration	2028	63	0.5714	0.5595
IEEE39	topology-guided	12-cycle duration	2029	63	0.5714	0.5595
IEEE39	topology-guided	12-cycle duration	2030	63	0.5714	0.5595
IEEE39	topology-guided	4-cycle duration	2026	63	0.8254	0.8162
IEEE39	topology-guided	4-cycle duration	2027	63	0.8254	0.8162
IEEE39	topology-guided	4-cycle duration	2028	63	0.8254	0.8162
IEEE39	topology-guided	4-cycle duration	2029	63	0.8254	0.8162
IEEE39	topology-guided	4-cycle duration	2030	63	0.8254	0.8162
IEEE39	random	12-cycle duration	2026	63	0.5714	0.5552
IEEE39	random	12-cycle duration	2027	63	0.5714	0.5552
IEEE39	random	12-cycle duration	2028	63	0.5714	0.5552
IEEE39	random	12-cycle duration	2029	63	0.5714	0.5552
IEEE39	random	12-cycle duration	2030	63	0.5714	0.5552
IEEE39	random	4-cycle duration	2026	63	0.8095	0.8065
IEEE39	random	4-cycle duration	2027	63	0.8095	0.8065
IEEE39	random	4-cycle duration	2028	63	0.8095	0.8065
IEEE39	random	4-cycle duration	2029	63	0.8095	0.8065
IEEE39	random	4-cycle duration	2030	63	0.8095	0.8065

Table S2 (continued). Primary fault-bus-localization fault-type and duration holdouts. Shift-A, Shift-B, and Shift-C withhold the stated fault type; Shift-D and Shift-E use the duration conditions stated above. Values are rounded to four decimals from the frozen phase25_stage2_shift_metrics.csv output.

System	Placement	Holdout condition	Seed	Events	Top-1	Macro-F1
IEEE123	topology-guided	C-G fault type	2026	183	0.0164	0.0008
IEEE123	topology-guided	C-G fault type	2027	183	0.0164	0.0006
IEEE123	topology-guided	C-G fault type	2028	183	0.0164	0.0006
IEEE123	topology-guided	C-G fault type	2029	183	0.0164	0.0006
IEEE123	topology-guided	C-G fault type	2030	183	0.0164	0.0007
IEEE123	topology-guided	B-C-G fault type	2026	183	0.2022	0.1414
IEEE123	topology-guided	B-C-G fault type	2027	183	0.2077	0.1443
IEEE123	topology-guided	B-C-G fault type	2028	183	0.1530	0.0972
IEEE123	topology-guided	B-C-G fault type	2029	183	0.2131	0.1496
IEEE123	topology-guided	B-C-G fault type	2030	183	0.1694	0.1118
IEEE123	topology-guided	A-B-C-G fault type	2026	183	0.0820	0.0425
IEEE123	topology-guided	A-B-C-G fault type	2027	183	0.0765	0.0425
IEEE123	topology-guided	A-B-C-G fault type	2028	183	0.0874	0.0527
IEEE123	topology-guided	A-B-C-G fault type	2029	183	0.0929	0.0443
IEEE123	topology-guided	A-B-C-G fault type	2030	183	0.0765	0.0432

System	Placement	Holdout condition	Seed	Events	Top-1	Macro-F1
IEEE123	topology-guided	12-cycle duration	2026	427	0.1124	0.0590
IEEE123	topology-guided	12-cycle duration	2027	427	0.1382	0.0728
IEEE123	topology-guided	12-cycle duration	2028	427	0.1499	0.0838
IEEE123	topology-guided	12-cycle duration	2029	427	0.1218	0.0639
IEEE123	topology-guided	12-cycle duration	2030	427	0.1358	0.0951
IEEE123	topology-guided	4-cycle duration	2026	427	0.0422	0.0093
IEEE123	topology-guided	4-cycle duration	2027	427	0.0445	0.0120
IEEE123	topology-guided	4-cycle duration	2028	427	0.0515	0.0126
IEEE123	topology-guided	4-cycle duration	2029	427	0.0609	0.0154
IEEE123	topology-guided	4-cycle duration	2030	427	0.0468	0.0079
IEEE123	random	C-G fault type	2026	183	0.0164	0.0007
IEEE123	random	C-G fault type	2027	183	0.0164	0.0008
IEEE123	random	C-G fault type	2028	183	0.0164	0.0005
IEEE123	random	C-G fault type	2029	183	0.0164	0.0013
IEEE123	random	C-G fault type	2030	183	0.0164	0.0005
IEEE123	random	B-C-G fault type	2026	183	0.2404	0.1998
IEEE123	random	B-C-G fault type	2027	183	0.2022	0.1372
IEEE123	random	B-C-G fault type	2028	183	0.2295	0.1708
IEEE123	random	B-C-G fault type	2029	183	0.2568	0.1929
IEEE123	random	B-C-G fault type	2030	183	0.1475	0.0897
IEEE123	random	A-B-C-G fault type	2026	183	0.0820	0.0434
IEEE123	random	A-B-C-G fault type	2027	183	0.0656	0.0363
IEEE123	random	A-B-C-G fault type	2028	183	0.0984	0.0560
IEEE123	random	A-B-C-G fault type	2029	183	0.0929	0.0557
IEEE123	random	A-B-C-G fault type	2030	183	0.1093	0.0625
IEEE123	random	12-cycle duration	2026	427	0.1288	0.0648
IEEE123	random	12-cycle duration	2027	427	0.1194	0.0727
IEEE123	random	12-cycle duration	2028	427	0.1452	0.0869
IEEE123	random	12-cycle duration	2029	427	0.1616	0.0984
IEEE123	random	12-cycle duration	2030	427	0.1194	0.0869
IEEE123	random	4-cycle duration	2026	427	0.0351	0.0083
IEEE123	random	4-cycle duration	2027	427	0.0398	0.0111
IEEE123	random	4-cycle duration	2028	427	0.0375	0.0111
IEEE123	random	4-cycle duration	2029	427	0.0398	0.0104
IEEE123	random	4-cycle duration	2030	427	0.0398	0.0076
IEEE39	topology-guided	C-G fault type	2026	27	0.1852	0.1081
IEEE39	topology-guided	C-G fault type	2027	27	0.1481	0.0919
IEEE39	topology-guided	C-G fault type	2028	27	0.1852	0.1278
IEEE39	topology-guided	C-G fault type	2029	27	0.3333	0.2421
IEEE39	topology-guided	C-G fault type	2030	27	0.2222	0.1439
IEEE39	topology-guided	B-C-G fault type	2026	27	0.3333	0.2364
IEEE39	topology-guided	B-C-G fault type	2027	27	0.3704	0.2614
IEEE39	topology-guided	B-C-G fault type	2028	27	0.4074	0.3694
IEEE39	topology-guided	B-C-G fault type	2029	27	0.2963	0.2111
IEEE39	topology-guided	B-C-G fault type	2030	27	0.3704	0.3046
IEEE39	topology-guided	A-B-C-G fault type	2026	27	0.2963	0.1561
IEEE39	topology-guided	A-B-C-G fault type	2027	27	0.4444	0.3254
IEEE39	topology-guided	A-B-C-G fault type	2028	27	0.2963	0.1556

System	Placement	Holdout condition	Seed	Events	Top-1	Macro-F1
IEEE39	topology-guided	A-B-C-G fault type	2029	27	0.3704	0.2296
IEEE39	topology-guided	A-B-C-G fault type	2030	27	0.3333	0.2068
IEEE39	topology-guided	12-cycle duration	2026	63	0.2857	0.2486
IEEE39	topology-guided	12-cycle duration	2027	63	0.2857	0.2578
IEEE39	topology-guided	12-cycle duration	2028	63	0.2540	0.1987
IEEE39	topology-guided	12-cycle duration	2029	63	0.2540	0.1971
IEEE39	topology-guided	12-cycle duration	2030	63	0.3016	0.2387
IEEE39	topology-guided	4-cycle duration	2026	63	0.2857	0.2175
IEEE39	topology-guided	4-cycle duration	2027	63	0.2540	0.2202
IEEE39	topology-guided	4-cycle duration	2028	63	0.2857	0.2715
IEEE39	topology-guided	4-cycle duration	2029	63	0.2381	0.2252
IEEE39	topology-guided	4-cycle duration	2030	63	0.3333	0.3236
IEEE39	random	C-G fault type	2026	27	0.2593	0.1753
IEEE39	random	C-G fault type	2027	27	0.1481	0.0833
IEEE39	random	C-G fault type	2028	27	0.2593	0.1814
IEEE39	random	C-G fault type	2029	27	0.2222	0.1537
IEEE39	random	C-G fault type	2030	27	0.3333	0.2357
IEEE39	random	B-C-G fault type	2026	27	0.4074	0.2920
IEEE39	random	B-C-G fault type	2027	27	0.3704	0.2880
IEEE39	random	B-C-G fault type	2028	27	0.3704	0.2852
IEEE39	random	B-C-G fault type	2029	27	0.3333	0.2620
IEEE39	random	B-C-G fault type	2030	27	0.3333	0.2343
IEEE39	random	A-B-C-G fault type	2026	27	0.4074	0.2598
IEEE39	random	A-B-C-G fault type	2027	27	0.4074	0.2663
IEEE39	random	A-B-C-G fault type	2028	27	0.3333	0.2032
IEEE39	random	A-B-C-G fault type	2029	27	0.3333	0.1772
IEEE39	random	A-B-C-G fault type	2030	27	0.3704	0.2254
IEEE39	random	12-cycle duration	2026	63	0.2857	0.2171
IEEE39	random	12-cycle duration	2027	63	0.3651	0.3217
IEEE39	random	12-cycle duration	2028	63	0.2222	0.1875
IEEE39	random	12-cycle duration	2029	63	0.2857	0.2115
IEEE39	random	12-cycle duration	2030	63	0.2857	0.2176
IEEE39	random	4-cycle duration	2026	63	0.2698	0.2183
IEEE39	random	4-cycle duration	2027	63	0.3175	0.3020
IEEE39	random	4-cycle duration	2028	63	0.2540	0.2013
IEEE39	random	4-cycle duration	2029	63	0.2698	0.2358
IEEE39	random	4-cycle duration	2030	63	0.2381	0.1849

S13 Bootstrap Sensitivity Analyses

Primary bus-by-condition Cartesian-product intervals and separately labeled sensitivity analyses; no significance selection is performed.

Table S3. Bootstrap sensitivity analyses. Primary=True identifies the bus-by-condition Cartesian-product analysis; other rows are sensitivity analyses. Values are rounded to four decimals from the frozen phase29_bootstrap_sensitivity.csv output.

System	Resampling method	Metric	Difference	CI lower	CI upper	Excludes 0	Primary
IEEE123	event_iid	top1	0.0167	0.0056	0.0270	True	False
IEEE123	event_iid	macro_f1	0.0182	0.0073	0.0288	True	False
IEEE123	event_iid	mean_hop	-0.0873	-0.1903	0.0094	False	False

System	Resampling method	Metric	Difference	CI lower	CI upper	Excludes 0	Primary
IEEE123	fault_bus_cluster	top1	0.0167	-0.0080	0.0462	False	False
IEEE123	fault_bus_cluster	macro_f1	0.0182	0.0022	0.0240	True	False
IEEE123	fault_bus_cluster	mean_hop	-0.0873	-0.3690	0.1407	False	False
IEEE123	condition_cluster	top1	0.0167	-0.0017	0.0351	False	False
IEEE123	condition_cluster	macro_f1	0.0182	-0.0009	0.0388	False	False
IEEE123	condition_cluster	mean_hop	-0.0873	-0.2594	0.1126	False	False
IEEE123	bus_x_condition_cartesian	top1	0.0167	-0.0180	0.0567	False	True
IEEE123	bus_x_condition_cartesian	macro_f1	0.0182	-0.0070	0.0337	False	True
IEEE123	bus_x_condition_cartesian	mean_hop	-0.0873	-0.4287	0.2523	False	True
IEEE39	event_iid	top1	0.0455	0.0190	0.0720	True	False
IEEE39	event_iid	macro_f1	0.0508	0.0262	0.0784	True	False
IEEE39	event_iid	mean_hop	-0.1397	-0.2413	-0.0413	True	False
IEEE39	fault_bus_cluster	top1	0.0455	-0.0148	0.1291	False	False
IEEE39	fault_bus_cluster	macro_f1	0.0508	-0.0079	0.0659	False	False
IEEE39	fault_bus_cluster	mean_hop	-0.1397	-0.3747	0.0476	False	False
IEEE39	condition_cluster	top1	0.0455	0.0211	0.0709	True	False
IEEE39	condition_cluster	macro_f1	0.0508	0.0272	0.0776	True	False
IEEE39	condition_cluster	mean_hop	-0.1397	-0.2498	-0.0349	True	False
IEEE39	bus_x_condition_cartesian	top1	0.0455	-0.0296	0.1397	False	True
IEEE39	bus_x_condition_cartesian	macro_f1	0.0508	-0.0205	0.0759	False	True
IEEE39	bus_x_condition_cartesian	mean_hop	-0.1397	-0.4317	0.0933	False	True

S14 Extended Physical Condition Evaluation

The authoritative extension contains 189 physical condition IDs across nine fault buses (1701 events). All events passed the final integrity audit, and each complete condition ID remains in one frozen outer fold.

Table S4. Frozen IEEE39 extended physical-condition configuration.

Field	Frozen value
Fault buses	3, 4, 15, 16, 18, 21, 23, 26, 29
Fault types	7 grounded types
Durations	4, 6, 12 cycles
Loads	90%, 100%, 110%
Rpn=Rng	0.1, 1, 20 Ω
Condition IDs	189
Events	1701
Outer folds	3; 63 condition IDs per fold
Integrity	1701/1701 PASS

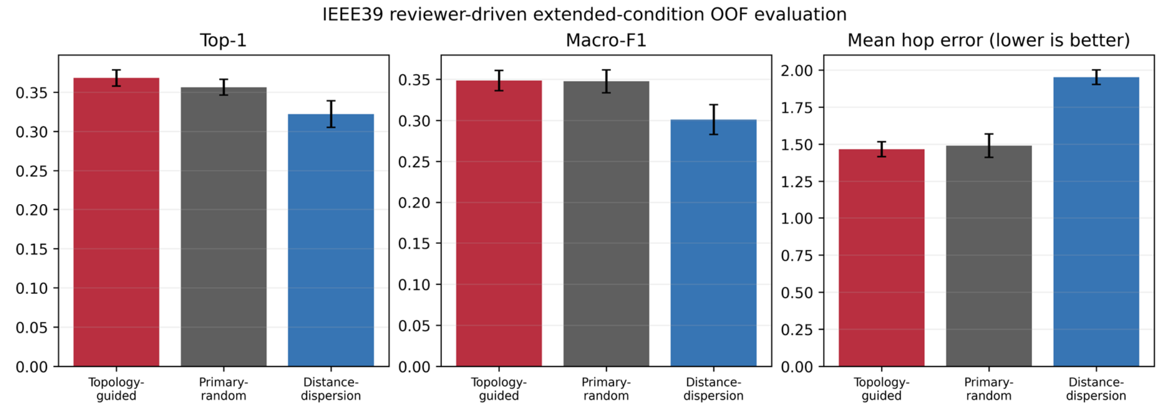

Figure S3. IEEE39 extended-condition pooled-OOF evaluation; error bars are SD across five complete seeds.

S15 Extended Seed Metrics and Paired Bootstrap

Table S5 reports the complete pooled-OOF seed-level metrics. Table S6 reports the paired bus-by-condition bootstrap. The paired intervals use 5000 independent bus and condition resamples evaluated on their Cartesian product.

Table S5. Extended pooled-OOF seed metrics.

Placement	Seed	Top-1	Top-3	Macro-F1	Mean hop	≤1 hop	≤2 hops
Topology-guided	2026	0.3757	0.7113	0.3499	1.4145	0.5897	0.7519
Topology-guided	2027	0.3527	0.6778	0.3368	1.4962	0.5444	0.7390
Topology-guided	2028	0.3792	0.6884	0.3681	1.4145	0.5720	0.7490
Topology-guided	2029	0.3651	0.7019	0.3393	1.4815	0.5473	0.7372
Topology-guided	2030	0.3686	0.7002	0.3485	1.5262	0.5432	0.7066
Primary-random	2026	0.3604	0.7266	0.3658	1.3774	0.5779	0.8001
Primary-random	2027	0.3715	0.7360	0.3575	1.4374	0.5608	0.7707
Primary-random	2028	0.3545	0.6878	0.3447	1.5379	0.5414	0.7331
Primary-random	2029	0.3469	0.6831	0.3311	1.5538	0.5262	0.7390
Primary-random	2030	0.3486	0.6808	0.3389	1.5450	0.5461	0.7213
Distance-dispersion	2026	0.3133	0.6343	0.2867	1.9559	0.4909	0.6208
Distance-dispersion	2027	0.3504	0.6696	0.3285	1.9371	0.5238	0.6126
Distance-dispersion	2028	0.3163	0.6249	0.2994	1.9924	0.4856	0.5838
Distance-dispersion	2029	0.3069	0.6020	0.2834	2.0006	0.4797	0.5897
Distance-dispersion	2030	0.3239	0.6367	0.3070	1.8771	0.5079	0.6238

Table S6. Topology-guided minus primary-random paired bootstrap.

Metric	Difference	95% CI	Crosses zero
Top1	+0.0119	[-0.0493, 0.0898]	Yes
MacroF1	+0.0009	[-0.0362, 0.0367]	Yes
mean_hop	-0.0238	[-0.2412, 0.1505]	Yes

S16 Load and Resistance Shift Evaluation

The shift models use only the specified training factor. Test load or resistance samples do not enter fitting, epoch selection, or hyperparameter selection.

Table S7. Load- and resistance-shift evaluation.

Family	Train	Test	Placement	Top-1	Top-3	Macro-F1	Mean hop
load shift	L100	L90	Topology-guided	0.1220	0.3693	0.0604	2.5570
load shift	L100	L110	Topology-guided	0.1280	0.3616	0.0583	2.1672
load shift	L100	L90	Primary-random	0.1203	0.3601	0.0514	2.5295
load shift	L100	L110	Primary-random	0.1295	0.3584	0.0624	2.1175
load shift	L100	L90	Distance-dispersion	0.1153	0.3503	0.0482	2.5877
load shift	L100	L110	Distance-dispersion	0.1189	0.3474	0.0399	2.1605
resistance shift	R_nominal	R_low	Topology-guided	0.4141	0.7407	0.3959	1.3231
resistance shift	R_nominal	R_high	Topology-guided	0.2776	0.5806	0.2671	1.7072
resistance shift	R_nominal	R_low	Primary-random	0.3891	0.7305	0.3760	1.3732
resistance shift	R_nominal	R_high	Primary-random	0.2649	0.5683	0.2544	1.8000
resistance shift	R_nominal	R_low	Distance-dispersion	0.3492	0.6399	0.3264	1.7686
resistance shift	R_nominal	R_high	Distance-dispersion	0.2011	0.4840	0.1885	2.3185

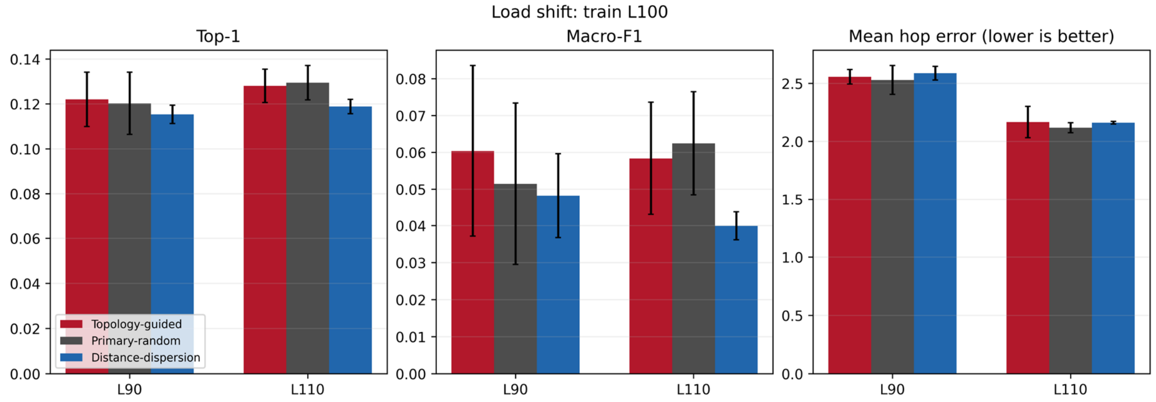

Figure S4. Load shift after training on L100; error bars are SD across five seeds.

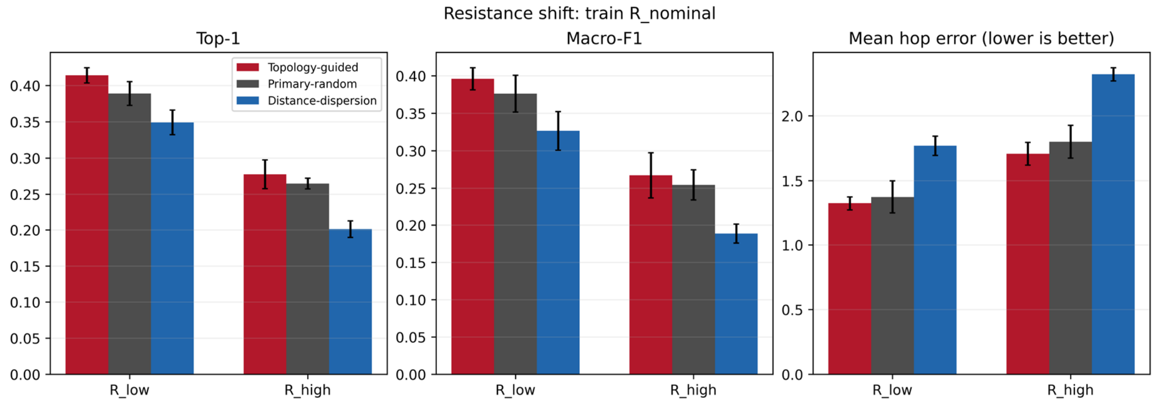

Figure S5. Resistance shift after training at Rpn=Rng=1 Ω; error bars are SD across five seeds.

S17 IEEE39 Complete 84 Layout Analysis

All 84 feasible three-sensor layouts were evaluated under the unchanged 189-event base protocol. The topology-guided layout is first on all three prespecified performance criteria.

Table S8. IEEE39 complete 84-layout analysis.

Metric	Topology mean	Rank	Better / equal / worse
Top-1	0.3905	1/84	0 / 1 / 83
Macro-F1	0.3579	1/84	0 / 1 / 83
Mean hop	1.5862	1/84	0 / 1 / 83

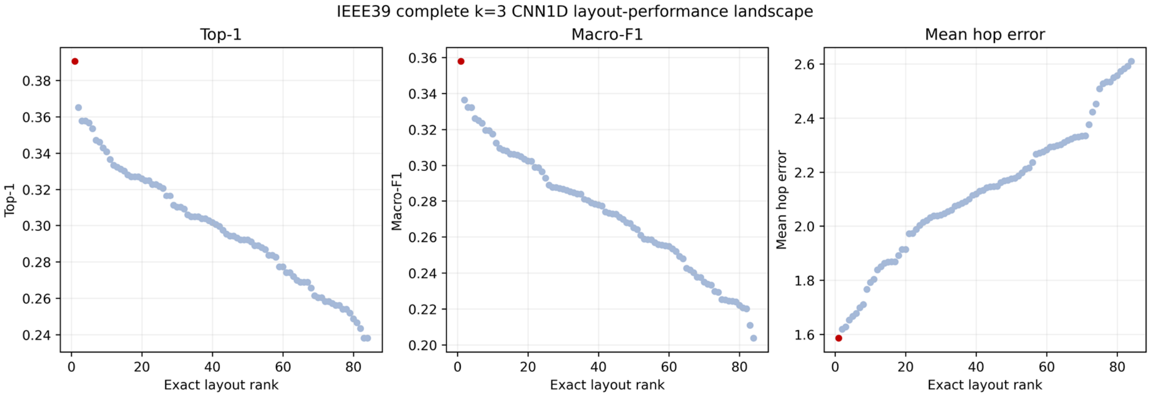

Figure S6. Complete IEEE39 84-layout CNN1D performance landscape under the frozen base protocol.

S18 Structural Layout Analysis

Topology-only structural metrics describe sensor dispersion and graph coverage. They are not classifier-performance tests.

Table S9. Structural layout metrics.

System	Named method	Minimum-pair rank/percentile	Mean-pair rank/percentile	Coverage-radius rank/percentile	Within-2 rank/percentile
IEEE39	topology-guided	1/84	1/84	1/84	1/84
IEEE123	topology-guided	100.00%	99.96%	99.11%	17.40%
IEEE39	distance-dispersion	25/84	30/84	77/84	27/84
IEEE123	distance-dispersion	95.23%	49.82%	51.75%	33.61%
IEEE39	degree-centrality	8/84	49/84	1/84	1/84
IEEE123	degree-centrality	74.14%	0.93%	74.61%	4.19%
IEEE39	primary-random	2/84	2/84	15/84	15/84
IEEE123	primary-random	74.14%	91.03%	51.75%	44.18%

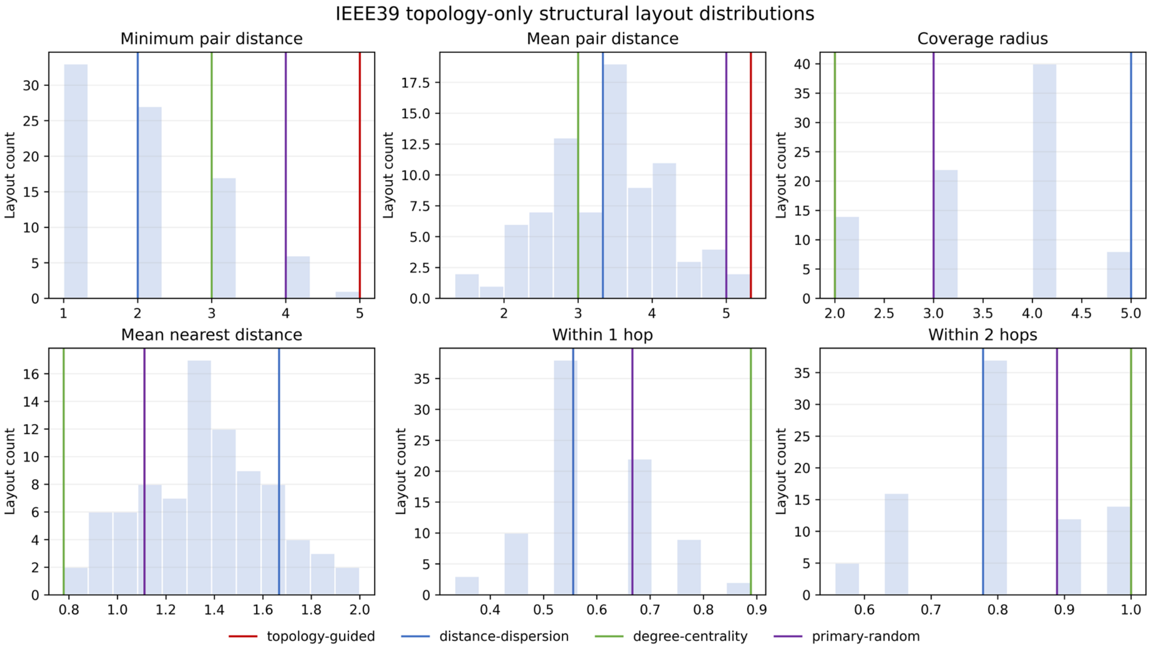

Figure S7. IEEE39 topology-only structural metric distributions across all 84 layouts.

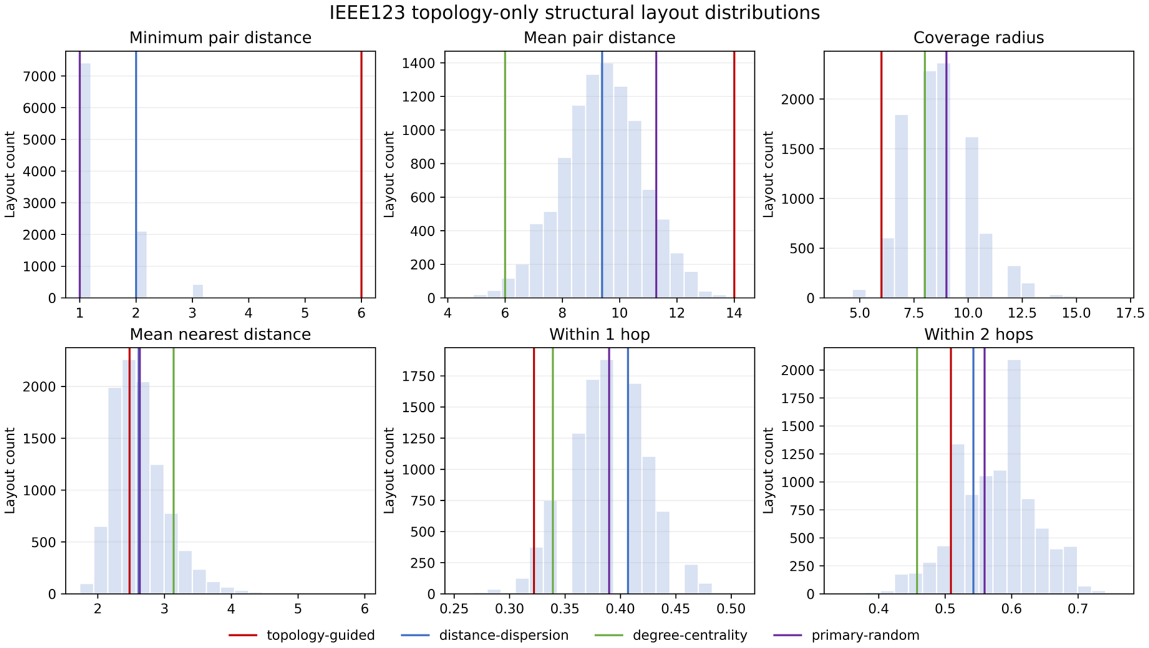

Figure S8. IEEE123 topology-only structural metric distributions for 10,000 frozen random layouts and four named layouts.
